# Selectively tuning ionic thermopower in all-solid-state flexible polymer composites for thermal sensing

**DOI:** 10.1038/s41467-021-27885-2

**Published:** 2022-01-11

**Authors:** Cheng Chi, Meng An, Xin Qi, Yang Li, Ruihan Zhang, Gongze Liu, Chongjia Lin, He Huang, Hao Dang, Baris Demir, Yan Wang, Weigang Ma, Baoling Huang, Xing Zhang

**Affiliations:** 1 Key Laboratory for Thermal Science and Power Engineering of Ministry of Education, Department of Engineering Mechanics, Tsinghua University, Beijing, 100084 China; 2 College of Mechanical & Electrical Engineering, Shaanxi University of Science and Technology, Xi’an, 710021 China; 3 Department of Mechanical and Aerospace Engineering, The Hong Kong University of Science and Technology, Clear Water Bay, Hong Kong SAR, China; 4 Department of Mechanical Engineering, Worcester Polytechnic Institute, 100 Institute Road, Worcester, MA 01609 USA; 5 Centre for Theoretical and Computational Molecular Science, The Australian Institute for Bioengineering and Nanotechnology, The University of Queensland, Brisbane, QLD 4072 Australia

**Keywords:** Thermoelectrics, Thermoelectric devices and materials

## Abstract

There has been increasing interest in the emerging ionic thermoelectric materials with huge ionic thermopower. However, it’s challenging to selectively tune the thermopower of all-solid-state polymer materials because the transportation of ions in all-solid-state polymers is much more complex than those of liquid-dominated gels. Herein, this work provides all-solid-state polymer materials with a wide tunable thermopower range (+20~−6 mV K^−1^), which is different from previously reported gels. Moreover, the mechanism of *p*-*n* conversion in all-solid-state ionic thermoelectric polymer material at the atomic scale was presented based on the analysis of Eastman entropy changes by molecular dynamics simulation, which provides a general strategy for tuning ionic thermopower and is beneficial to understand the fundamental mechanism of the *p*-*n* conversion. Furthermore, a self-powered ionic thermoelectric thermal sensor fabricated by the developed *p*- and *n*-type polymers demonstrated high sensitivity and durability, extending the application of ionic thermoelectric materials.

## Abstract (web summary)

Though high ionic thermopower and p-n conversion has been realized in liquid ionic thermoelectric materials, achieving similar performance in solid-state polymer materials remains a challenge. Here, the authors report all-solid-state thermoelectric polymer composites with tunable ionic thermopower.

## Introduction

Thermal sensors (TS) play a crucial role in various areas such as aerospace, industry, agriculture, biomedical sensing, monitoring of microprocessors, and environmental temperature measurements^1,2^. Specifically, TS for heat detection must be accurate, efficient, scalable, and cost-effective. Thermoelectric (TE) technology is a powerful technology that can directly convert the temperature differences to the electric voltage based on the Seebeck effect with a lower level of vibration and noise, less maintenance necessities, precise temperature control and high reliability^3–5^. The traditional TE materials based on inorganic semiconductors^5^ and semi-metals^6,7^ have been extensively investigated, however, they still suffer from low thermopower (~100–200 μV K^−1^), less flexibility, low Earth abundance and substantial production cost which usually requires the integration of thousands of thermoelements connected in series, rendering them not conducive to large-scale industrial applications^8–10^.

Alternatively, high ionic thermopower or Seebeck coefficient (*S*_*i*_) was found in the ionic thermoelectric (*i*-TE) materials based on the Soret effect, providing a promising route to develop high-performance TE materials^11–13^. For example, polyethylene oxide (PEO)/NaOH/H_2_O solution^14^ exhibited a thermopower of 10 mV K^−1^, cellulose-polystyrene sulfonate sodium (NFC-PSSNa) had a thermopower of 8.4 mV K^−1^ at 100% relative humidity (RH)^15^ and a synergistic KCl/ferro/ferricyanide (FeCN_6_^4–/3–^) gelatin offered a high thermopower of 17.0 mV K^−1^, which is much higher than pure solutions of KCl (0.04 mV K^−1^) and FeCN_6_^4–/3–^ (1.4 mV K^−1^)^16^. These liquid-dominated gels are found to exhibit larger thermopower than that of the liquid solution alone. In addition to the thermal transport difference between cations and anions, it is suspected that the enhancement on thermopower is also attributed to the Eastman entropy which was induced by more complex interatomic interactions among ions, polymers and surrounding media^8,16^. However, the quantitative relationship between complex interactions and the Eastman entropy change of cations and anions in all-solid-state polymer *i*-TE materials has not been demonstrated at the atomic scale to this end.

Moreover, as most polymer-based *i*-TE materials show positive thermopowers, several efforts have been attempted to investigate the *p*-*n* conversion in liquid *i*-TE materials, i.e 1-ethyl-3-methylimidazolium acetate (EMIm:Ac)/H_2_O solution^17^, iodide/triiodide (I^−^/I_3_^−^) thermogalvanic cell^18^ liquid systems and 1-ethyl-3-methylimidazolium bis(trifluoromethylsulfonyl)imide (EMIM:TFSI) based liquid-dominated gels^9^. However, few reports have reported the *p*-*n* conversion in the all-solid-state *i*-TE polymer materials so far. Particularly, it’s very significant to convert *p*-type all-solid-state *i*-TE material to *n*-type when fabricating high-performance *i*-TE modules. Compared with liquids and liquid-dominated gels, all-solid-state *i*-TE polymer materials provide unique merits, such as no leakage, high mechanical strength, ease in fabrication, and good durability. In addition, it’s almost impossible to deposit a uniform metal contact layer on these liquids and gels as the liquid will be evaporated under a high vacuum or the liquid could result in a discontinuous contact layer due to rapid cooling, which seriously limited the practical applications. Despite the benefits of all-solid-state *i*-TE materials, the lacking of efficient negative thermopower still hindered the development of all-solid-state *i*-TE based devices^9,19^. It is very challenging to selectively tune the positive thermopower to negative of all-solid-state *i*-TE polymer materials because all-solid-state polymer systems are much more complex than those liquids and their performances are affected by various factors. Especially, the transportation of ions in the all-solid-state polymer is fundamentally different from that in liquid-dominated gels or liquids, in which the movement of ions relies on the interaction with the polar group of polymers combining with the segmental motion of polymer chains instead of the straightforward diffusion in the liquid-dominated gels^20,21^. Unluckily, fundamental studies into the mechanism of ionic thermopower conversion in all-solid-state polymers are lacking, and there is rarely a universal strategy reported to convert *p*-type thermopower to *n*-type, seriously impeding the fundamental understanding and the practical application of *i*-TE polymer materials-based devices.

To address these issues, our work successfully achieved the conversion from *p*-type all-solid-state polyvinylidene fluoride-hexafluoropropylene (PVDF-HFP)/sodium bis(trifluoromethylsulfonyl)imide (NaTFSI)/propylene carbonate (PC) [PVDF-HFP/NaTFSI/PC (PhNP)] to *n*-type with a wide tunable ionic thermopower range (+20 to −6 mV K^−1^) by regulation of the transportation of Na^+^ ions. The mechanism of the *p*-*n* conversion based on analysis of Eastman entropy change in all-solid-state *i*-TE polymer material was presented at the atomic level. The molecular dynamics (MD) simulation results found that the conversion in thermopower is strongly related to Eastman entropy change in the charge type of the ions, as the entropy change of Na^+^ cations is higher than that of TFSI^−^ anions in the *p*-type PhNPs and vice versa, which provides a general strategy for tuning ionic thermopower in all-solid-state polymers. Besides a high-performance planar *i*-TE generator (*i*-TEG), we further demonstrated an *i*-TE TS with high sensitivity, extending the application of *i*-TE materials.

## Results

### Thermoelectric properties of *p*-type PhNPs

**Fig. 1:**
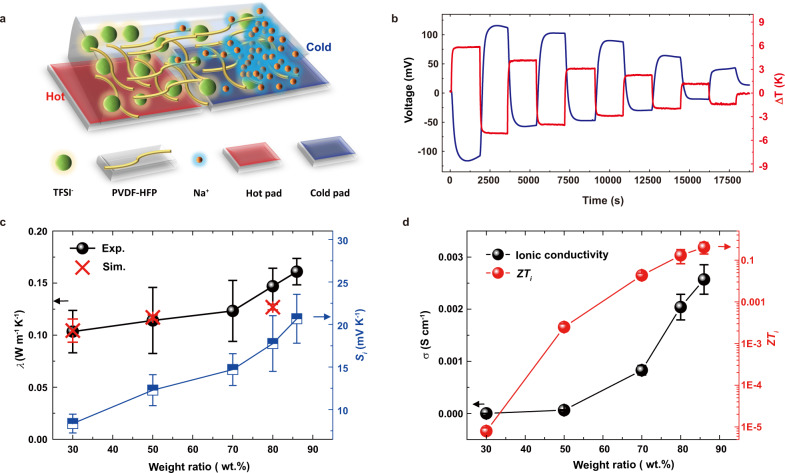
The thermoelectric performance of the *p*-type PhNP. **a** The diagrammatic illustration of the *p*-type (Na^+^ cations dominate thermodiffusion) PhNP *i*-TE materials. **b** The plot of the measured Δ*V*_*i*_ −Δ *T* curves of PhNP-86. **c** The measured thermopower, thermal conductivity together with simulation results of PhNPs. **d** The measured ionic conductivity and figure of merit *ZT*_*i*_ of each PhNPs.

The developed flexible all-solid-state *p*-type *i*-TE PhNP composites (Supplementary Fig. 1) are composed of PVDF-HFP, NaTFSI salt and low-molecular-weight PC, in which the weight ratios of NaTFSI/PC to overall composite range from 30 to 86 wt.%, which are assigned as PhNP-30 to PhNP-86, respectively and the preparation procedures are illustrated in Supplementary Information. The ionic thermopower was measured through a home-made in-plane setup (Supplementary Fig. 2) which was calibrated with the reported materials in the previous reports^8,9,14^. To be noticed, the starting hot and cool ends of PhNPs were electrically connected to the positive and negative poles of a voltmeter, respectively. When a temperature difference (Δ*T* = + 6 K) was applied, a negative thermal voltage of PhNP-86 was produced (Fig. 1b). Once alternating of the hot and cool sides (Δ*T* = −6 K), the generated TE voltage instantly turned to the positive direction correspondingly, demonstrating rapid and reversible thermal response behavior. The observations suggested a higher concentration of positive Na^+^ cations accumulated at the cool side as illustrated in Fig. 1a. It is also implied the thermal mobility of Na^+^ ions was larger than that of TFSI^−^ anions, belonging to a *p*-type *i*-TE material^9,16^. Besides, the higher the temperature difference, the larger thermal voltage was generated by PhNPs, exhibiting high capability in thermal-intensity sensing. The *S*_*i*_ of each PhNPs was obtained by fitting the slope of the measured Δ*V*_*i*_ − Δ*T* curves (Supplementary Fig. 3) according to Eq. (1)

1 $${S}_{i}=\frac{V({T}_{H})-({T}_{C})}{{T}_{H}-{T}_{C}}$$

where *V*(*T*_H_) and *V*(*T*_C_) correspond to the voltage of the hot electrode at temperature *T*_H_ and the cold electrode at temperature *T*_C_, respectively.

Interestingly, the thermopower of the PhNPs gradually increased with the increasing weight ratio of NaTFSI/PC and reached around 20 ± 4 mV K^−1^ of PhNP-86 (Fig. 1c) at 68% RH, demonstrating high performance compared to the recently reported works, such as 11 mV K^−1^ of PEO/NaOH/H_2_O^14^ and 13 mV K^−1^ of PVDF-HFP/EMIM:TFSI/polyethylene glycol (PEG)^9^. The ionic conductivity (*σ*_*i*_) of each PhNPs was measured by the electrochemical impedance method^22^ and the corresponding measured impedance spectra were plotted in Supplementary Fig. 4. Similarly, the ionic conductivity was improved from the PhNP-30 of ~4 × 10^−7^ S cm^−1^ to the highest value in PhNP-86 of ~2.6 × 10^−3^ S cm^−1^ (Fig. 1d) which is near to that of the pure liquid NaTFSI/PC (6 × 10^−3^ S cm^−1^). At the high weight ratio of NaTFSI/PC, the PhNP-86 formed a relatively homogeneous and cross-linked porous structure as observed from the cross-section view in Supplementary Fig. 5, which can improve the diffusion coefficient of ions and facilitate the ions transport^23,24^. In addition, the thermal conductivity (*λ*_*i*_) of the PhNPs was measured through the Hot-disk method (Supplementary Fig. 6) and it exhibited an increasing trend from 0.1 to 0.16 W m^−1^ K^−1^ with increasing the weight ratio of NaTFSI/PC. The calculated thermal conductivities from MD simulations for PhNP-30, -50, -80 were around 0.1, 0.117 and 0.127 W m^−1^ K^−1^, respectively, which matched well with experimental data (Fig. 1c) and were consistent with the thermal conductivity range (0.1–0.3 W m^−1^ K^−1^) of amorphous materials^25,26^. Moreover, the TE performance of PhNP-86 with different concentrations of NaTFSI (0.2, 0.5, 1.0, and 2.0 M) was also studied, where the weight ratio of NaTFSI/PC to the overall PVDF-HFP/NaTFSI/PC was kept as 86 wt.%. Both the ionic thermopower and conductivity were found to gradually increase and obtained at an optimum value of 1.0 M sample (Supplementary Fig. 7a). However, the *S*_*i*_ and *σ*_*i*_ decreased when the concentration of NaTFSI was over 2.0 M, because higher concentration was easy to form ion clusters and it generated high viscosity which reduced the number of free ions and slowed down the ion diffusion process^27,28^. The thermal conductivity slightly increased with the increasing concentration of NaTFSI and the value ranged from 0.15 to 0.17 W m^−1^ K^−1^ (Supplementary Fig. 7b). The dimensionless ionic figure of merit *ZT*_*i*_, which is defined as *ZT*_*i*_ = *S*_*i*_^2^*σ*_*i*_*T*/*λ*_*i*_, is used to characterize the performance of *i*-TE materials^29^. A high thermopower, ionic conductivity and low thermal conductivity of the developed PhNPs greatly contributed to achieving a high *ZT*_*i*_. As a result, the *ZT*_*i*_ of PhNP-86 with 1.0 M NaTFSI obtained a maximum value of over 0.2 at 298 K, as shown in Fig. 1d and Supplementary Fig. 7b, which is higher than some recently reported *i*-TE materials such as PEO/NaOH (0.014)^14^, PSSNa (0.013)^30^ and PVDF-HFP/EMMI:TFSI (0.007)^9^.

### Thermoelectric properties analysis

**Fig. 2:**
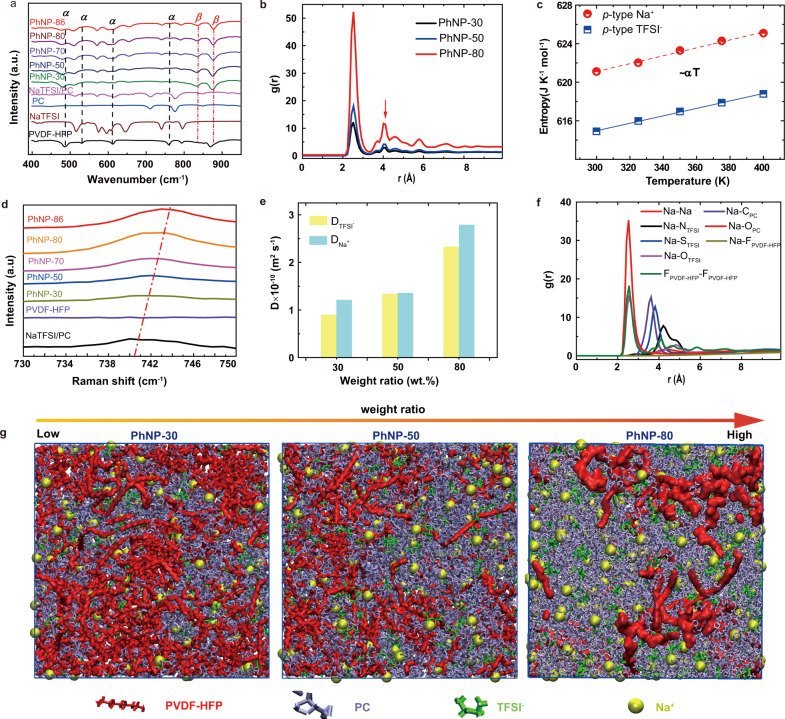
The structural characterization and atomic-level interaction in PhNPs. **a** FTIR spectra with a range of 400–950 cm^−1^, **b** the calculated RDFs of the PhNP-30, -50 and -80 samples, **c** the calculated entropy change of cations and anions in PhNP-86, **d** the Raman spectra with a range of 733–746 cm^−1^ of each PhNPs, **e** the diffusion coefficient of Na^+^ and TFSI^−^ in PhNP-30, -50 and -80 samples, **f** the RDF of each interaction among ions and PVDF-HFP of PhNP-50 and **g** the snapshots of the PhNP-30, -50 and -80 samples in the MD simulation model.

In this work, the weight ratio of NaTFSI/PC is found to have a significant effect on both the thermopower and ionic conductivity of the PhNP. As suggested by a recent work^16^, the generated thermopower can be described by Eq. (2):

2 $${S}_{td}=\frac{{\sum }_{i}{q}_{i}{n}_{i}^{0}{\hat{S}}_{i}{D}_{i}}{{\sum }_{i}{q}_{i}^{2}{n}_{i}^{0}{D}_{i}}$$

where *q*, *n*^*0*^_*i*_, *i*, *Ŝ* and *D*_*i*_ represent charges, concentration, ion species, the Eastman entropy and diffusion coefficient, respectively. *S*_*td*_ is strongly associated with the change in *Ŝ*_*i*_ and mass transport difference between positive and negative ions at a given temperature difference. Previous work^14,16,31^ suggested that the interaction between ions and polymer matrix was considered to have a substantial impact on the Eastman entropy. To understand the interaction between PVDF-HFP and ions, the Fourier transform infrared spectroscopy (FTIR) characterization from 400 to 950 cm^−1^ of each PhNPs was conducted, as shown in Fig. 2a. Upon incorporation of NaTFSI/PC into PVDF-HFP, the intensity of the crystalline phase (*α*-phase) of PVDF-HFP, which is located at 980 cm^−1^ (—CF_2_ and —CC symmetric stretching), 796 cm^−1^ (—CF_3_ stretching), 762 cm^−1^ (—CH_2_ rocking), 614 cm^−1^ (—CF_2_ bending and CCC skeletal), and 531 cm^−1^ (—CF_2_ wagging)^32,33^, gradually decreased and peaks of 614 and 762 cm^−1^ even disappeared at higher concentration of NaTFSI/PC. Meanwhile, the peak at 879 cm^−1^ belonging to the amorphous phase (*β*-phase) became dominant and another *β*-phase peak at 841 cm^−1^ appeared. On one hand, as the ion conduction in polymer occurs mainly in the amorphous region^21,34^, the increased amorphous region (*β*-phase) of PVDF-HFP is favored to improve ion mobility. On the other hand, the formation of the *β*-phase is attributed to the interaction between the dipole moment of polymer and ions in which a large spontaneous polarization can be generated^35,36^.

Moreover, to gain molecular-level insights into the interactions between the ions and polymer, and the entropy change, we performed all-atom MD simulations and the details of the calculation method are illustrated in Supplementary Information. Figure 2g shows the simulation snapshots taken from the PhNP-30, -50 and -80 samples, where the PVDF-HFP chains, PC small molecules, TFSI^−^ anions and Na^+^ cations distribute uniformly in the simulations cell. With the increasing weight ratio of NaTFSI/PC, the intertwined chains of PVDF-HFP tend to spread and the interactions among chains/ions could be changed accordingly. The scanning electron microscope (SEM) also found that the packed PVDF-HFP was quickly changed to the porous structure of PhNPs and the size of quasi-spherical grains of the polymer chains gradually decreased (Supplementary Fig. 5a–j), which are consistent with the built MD model (Fig. 2g). The calculated radial distribution functions (RDFs) between fluorine (F) atoms in PVDF-HFP showed that the amplitude of the former peak at 2.5 Å increased from the sample PhNP-30 to PhNP-80 (Fig. 2b), indicating more structural heterogeneity in the sample with the increasing weight ratio of NaTFSI/PC. The RDF is defined as the probability of finding a pair of atoms at a particular separation compared to that of a random distribution at the same density. It also suggests that the local density of the PVDF-HFP chains in the PVDF-HFP rich area will be much higher than the average value over the whole simulations and explain the large variation in peak height. Meanwhile, we also observed a peak at 4.2 Å gradually loomed with the increasing weight ratio of NaTFSI/PC. This peak strongly implies that partial interaction at 2.5 Å converts to weak force among PVDF-HFP separated chains in PhNP-80, which is consistent with the emerging phase in the experimental finding in Fig. 2a. Furthermore, the entropies of both the Na^+^ cations and TFSI^−^ anions were predicted by the MD simulations^37,38^ (detail in Supplementary Information). They almost exhibited a linear relationship with temperature (Fig. 2c), which is consistent with the second law of thermodynamics. The calculated slope of entropy change of Na^+^ ions (0.040) is larger than that of TFSI^−^ ions (0.038) under the same temperature difference, suggesting a larger driving force of Na^+^ ions from entropy change and thus enhancing the thermopower.

Another important factor to improve thermopower is to enlarge mass transport differences of cations and anions which usually enhance potential imbalance. From Raman analysis (Supplementary Fig. 8), it is clear to find the intensity of the peak located at 740–741 cm^−1^, which is assigned to TFSI^−^ anions, gradually becomes more predominant, confirming more amount of NaTFSI/PC was introduced. Meanwhile, the peak position of TFSI^−^ exhibited a shift to a larger wavelength (743–744 cm^−1^) direction (Fig. 2d), which indicates that more amount of the TFSI^−^ anions formed contact ion pairs^28,39^. The vibrational spectrum of contact ion pairs can be influenced by both ionic interaction and conformational effects. The contact ion pairs tend to form NaTFSI, [Na(TFSI)_2_]^−^, [Na(TFSI)_3_]^2−^ and [Na(TFSI)_4_]^3−^ complexes^40^ as illustrated in Supplementary Fig. 9a. It’s speculated that a larger number of TFSI^−^ anions is involved in the complexes than Na^+^ cations which might be responsible for enlarging the mass transport difference between Na^+^ and TFSI^−^. In addition, the analysis of MD simulation results showed that the diffusion coefficient of Na^+^ cations (2.79 × 10^−10^ m^2^ s^−1^) was substantially higher than that of TFSI^−^ anions (2.32 × 10^−10^ m^2^ s^−1^) of PhNP-80 (Fig. 2e) which was consistent with the experimental results by Pulsed-field gradient nuclear magnetic resonance^41^. Moreover, in a negatively charged polymer network, the cations usually show a higher diffusion coefficient according to the experimental^16,42^ and computational analysis^43,44^. As a small portion of cations tend to condensate along the negative sites of polymer chains according to the counterion condensation limiting laws^45^, where these immobilized Na^+^ around the PVDF-HFP chains generate frictional resistance on the free TFSI^−^ anions. At the same time, the formed electrostatic field surrounding the PVDF-HFP enables to govern Na^+^ ions transport along the direction from the hot to cold side (Supplementary Fig. 9b). This disparity facilitates the transport of Na^+^ ions and impedes the movement of TFSI^−^ ions^46^.

The increased thermal conductivity of PhNPs was ascribed to the enhanced heat transport contribution from electrostatic interactions between ions and the surrounding environment in the system with an increasing weight ratio of NaTFSI/PC. To better understand the interatomic interactions, the RDFs of Na-O_PC_, Na-O_TFSI_, Na-N_TFSI_, Na-S_TFSI_, Na-C_PC_, and F_PVDF-HFP_-F_PVDF-HFP_ pairs of PhNP-50 as a typical example were studied (Fig. 2f). It showed that a peak position at around 2.6 Å between the charged pairs (Na-Na, Na-O_PC_, and Na-O_TFSI_) which suggested the distance of the charged pairs is more likely to be closer than the other pairs, i. e. Na-F_PVDF-HFP_, Na-C_PC_, and Na-S_TFSI_ pairs due to the stronger electrostatic interactions among the former charged pairs. As the weight ratio of NaTFSI/PC increased, they provided larger proportional electrostatic interactions and more thermal pathways for phonon transport due to the transition from weak Van der Waals interaction to strong Coulomb interactions.

### Converting *p*-type PhNP to *n*-type

**Fig. 3:**
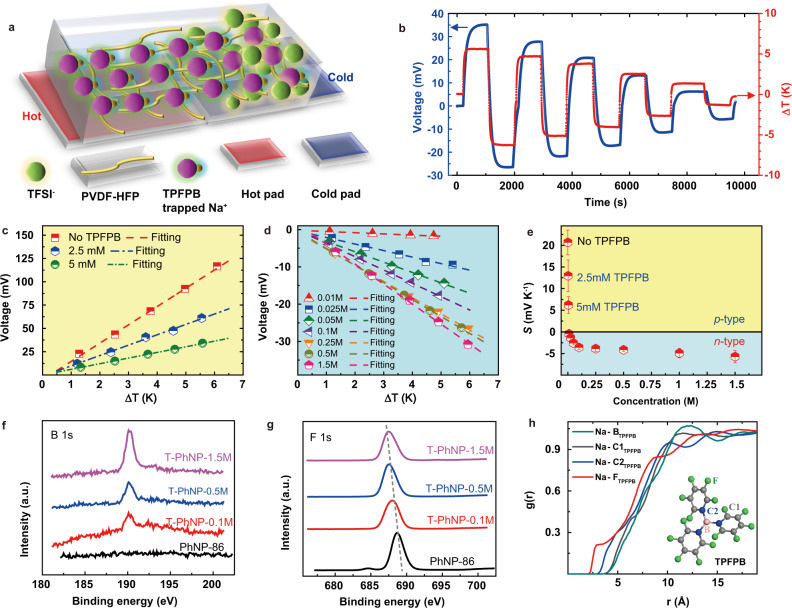
The conversion from *p*-type PhNP to *n*-type T-PhNP. **a** The schematic of ion transport of *n*-type (TFSI^−^ anions dominate thermodiffusion) T-PhNP *i*-TE materials. **b** The generated voltage under a series of temperature differences of T-PhNP-1.5 M. **c**, **d** The plot of fitting curves of Δ*V*_*i*_-Δ*T* and **e** the calculated thermopower of each T-PhNPs. XPS characterization of **f** B (1 s) and **g** F (1 s) peak of T-PhNPs. **h** The radial distribution function of Na^+^ cations and the atoms of TPFPB molecules.

Regulating ion transportation is an effective way to tune the sign of thermopower. Here, tris(pentafluorophenyl)borane (TPFPB) molecule was incorporated to the *p*-type PhNP-86, which could provide a negative-charged enriched environment in the composite. After introducing 1.5 M TPFPB, the generated voltage exhibited a positive value (red line, Fig. 3b) with a Δ*T* (+6 K) at the starting state, which was opposed to the sign of generated voltage of *p*-type PhNP-86. The reversing sign of voltage suggested that a higher concentration of TFSI^−^ ions was accumulated at the cool side (Fig. 3a), indicating TFSI^−^ anions dominated the thermodiffusion process, realizing the conversion from *p*-type PhNPs to *n*-type. To comprehensively study the influence of TPFPB on tunning thermopower, a series of TPFPB concentrations from 2.5 mM to 1.5 M were introduced into *p*-type PhNP-86 and assigned as T-PhNP-*x*M, where *x* is the concentration of TPFPB. The original porous morphologies gradually became more compact structures, and the pores were even filled at high concentrations of the TPFPB (Supplementary Fig. 10a–c). The energy dispersive X-ray spectroscopy (EDS) mapping of T-PhNP-0.5 M (Supplementary Fig. 10d) demonstrated the distribution of each element of the fabricated *i*-TE materials. Specifically, the boron (B) element only coming from TPFPB was found on the surface of T-PhNPs, confirming that the T-PhNPs composites were successfully formed.

The thermopower of the T-PhNP-2.5 mM and T-PhNP-5 mM decreased to 12.95 and 6.2 mV K^−1^, respectively, indicating the thermodiffusion of cations was impeded as shown in Fig. 3c. Interestingly, the thermopower of T-PhNPs-0.01 M is close to zero (Fig. 3d), representing the thermodiffusion of cations is nearly close to anions. Moreover, when the concentration of TPFPB exceeded this critical point, the thermopower became more negative with containing more amount of TPFPB (Fig. 3d, e and Supplementary Fig. 11), strongly suggesting the thermodiffusion of anions was easier than cations of the T-PhNPs. And the maximum value reached around −6 ± 1 mV K^−1^ of T-PhNP-1.5 M, which is comparable with those of the reported organic *n*-type *i*-TE materials so far, such as the PVDF-HFP/EMIM:TFSI (−4 mV K^−1^)^9^, tetrachloro-perylene bisimide (4Cl-PBI) (−3.02 mV K^−1^)^47^ at room temperature. From the X-ray photoelectron spectroscopy (XPS) analysis (Supplementary Fig. 12 and Fig. 3f, g), it is clear to find that the intensity of the peak located at 191 eV gradually became stronger (Fig. 3f), which was assigned to B atoms, suggesting more amount of TPFPB was introduced. Moreover, the binding energy of F1s tends to shift from 688 to 686 eV upon adding TPFPB. Since the interaction between F and Na^+^ could change the energy status of F of TPFPB, making the binding energy towards the Na-F of which the binding energy is around 684 eV^48^. In addition, the built MD model of T-PhNP-1.5 M and the calculated RDFs between Na^+^ and atoms of TPFPB molecules from MD simulations are shown in Supplementary Fig. 13a and Fig. 3h, respectively. The RDFs indicate that the F atom in TPFPB molecules has the strongest interaction with Na^+^ ions. It’s speculated that the incorporation of TPFPB molecules not only traps Na^+^ ions but also breaks up the interactions between Na^+^ and PVDF-HFP and disturbs the transport path of Na^+^ ions along the PVDF-HFP polymer chain. Besides, MD results suggested that the slope of entropy change with temperature of TFSI^−^ anions (0.0365) is larger than that of Na^+^ cations (0.0317) in *n*-type T-PhNP-1.5 M (Supplementary Fig. 13b, c). It indicates that the addition of TPFPB makes a larger change in entropy and creates larger driving force for TFSI^−^ anions than Na^+^ cations, which is opposite to that of *p*-type PhNPs, realizing the transition from *p*-type to *n*-type of the developed *i*-TE materials. Furthermore, the measured ionic conductivities of *n*-type T-PhNP-0.5 M and -1.0 M were 1.5 × 10^−3^ S cm^−1^ and 9.33 × 10^−4^ S cm^−1^, respectively, (Supplementary Fig. 14). The introduced TPFPB not only trapped parts of Na^+^ ions, but also the formed dense structure may improve crystallinity, reducing the number and mobility of ions. The thermal conductivities of T-PhNP-0.5 M and -1.0 M at 298 K are 0.165 W m^−1^ K^−1^ and 0.185 W m^−1^ K^−1^, respectively, gradually increasing after introducing TPFPB. The figure of merit of *ZT*_*i*_ for *n*-type T-PhNP-0.5 M and -1.0 M are 0.0046 and 0.0037, respectively.

For practical application, the performance of both *p*- and *n*-type *i*-TE materials under different RH was studied. The ionic thermopower of the *p*-type PhNP-86 sample increased from 8.12 mV K^−1^ (40% RH) to 26.62 mV K^−1^ (90% RH) as shown in Supplementary Fig. 15a. As the porous structure of PhNP-86 and the hydrophilic nature of sodium salt tend to absorb water from the moisture environment, the absorbed water could fill the space in the polymer matrix^49,50^ and help improve the dissociation of the NaTFSI counter-ions by weakening the electrostatic potential. However, the thermopower of the *n*-type T-PhNP-1.0 M exhibited a decreasing tendency with the increasing humidity (Supplementary Fig. 15b). It’s suspected that the absorbed water may interact with TPFPB at high humidity^51,52^, which may block the ions transportation pathway and reduce ions’ mobility. In addition, the durability of the *n*-type T-PhNP-1.0 M sample was further investigated. It’s clear to find the thermopower of T-TPFPB-1.0 M remained at an almost constant value (−5 to −6 mV K^−1^) for over more than 18 days at 68% RH, demonstrating strong durability (Supplementary Fig. 15c and d). Moreover, it’s crucial to deposit a uniform metal contact layer on *i*-TE materials when fabricating *i*-TE modules. But it’s almost impossible to deposit a uniform metal contact layer on the surface of liquids or gels under a high vacuum condition^53^. Interestingly, our developed all-solid-state *n*-type T-PhNP can easily achieve the uniform deposition of an Au metal layer by the physical vacuum evaporation (PVD) method attributed to the solid physical features, as shown in Supplementary Fig. 16a, c. For comparison, the reported PVDF-HFP/EMIM:TFSI gel^9^ was selected through the same PVD process. Unluckily, it formed a discontinuous Au layer and the color of the surface became black, representing such liquid-dominated gels cannot survive when involved in the high vacuum process (Supplementary Fig. 16b, d). Clearly, it is a significant step to prove that the possibility of *i*-TE materials can be further extended to develop micro/nano ionic TE-based devices by standard fabrication process in the semiconductor industry.

### The prototype of all-solid-state *i*-TE devices

**Fig. 4:**
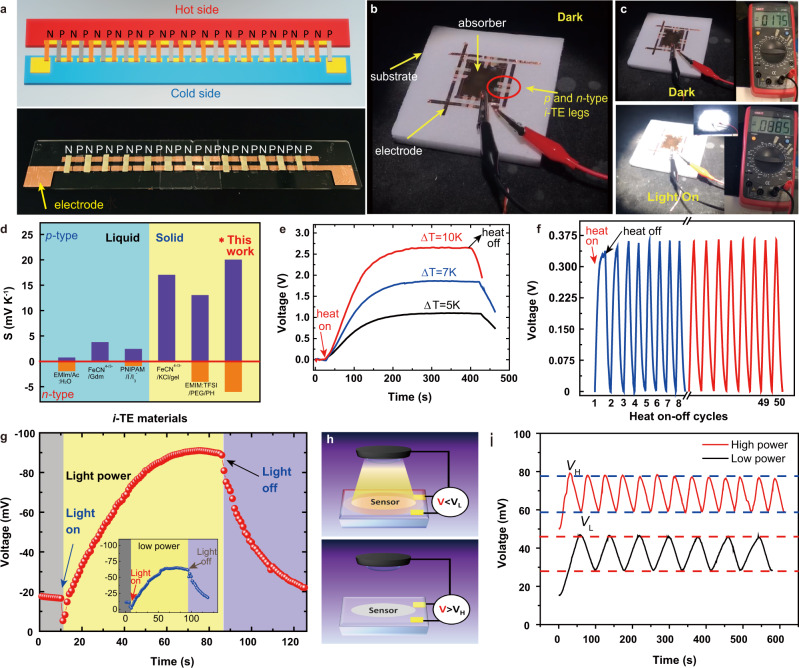
The performance of the prototype of all-solid-state *i*-TE devices. **a** The digital photo of the design and the fabricated in-plane *i*-TEG with 13 pairs of *p*-*n* thermocouples. **b**, **c** The demonstration of ionic-thermoelectric thermal sensors. **d** Comparison of the thermopower range for the recently reported *i*-TE materials realizing *p*-*n* conversion^9,17,18^. **e** The thermoelectric voltage (*V*) *vs* time (*t*) curves of the fabricated *i*-TEG under various ∆*T*. **f** The 50-cycles heat on/off stability test of the as-fabricated *i*-TEG under 1.5 K. **g** The performance of ionic-thermoelectric light-induced thermal sensors. **h** The demonstration and (**i**) performance of a self-powered LiTS system that can automatically control the lamp.

A planar *i*-TEG was fabricated, which included 13 pairs of *p*-type PhNP-86 and *n*-type T-PhNP-1.5 M films shown in Fig. 4a. The produced thermal voltage of the *i*-TEG reached over ~2.6 V at ∆*T* = 10 K in about 200 s (Fig. 4e) in the air, indicating good thermal response property. The overall thermopower of this *i*-TEG reached as high as 0.265 V K^−1^, which is near to the theoretical sum of the thermopower of PhNP-86 and T-PhNP-1.5 M TE legs (0.338 V K^−1^) (Supplementary Fig. 17). To the best of our knowledge, the developed PhNP/T-PhNP thermocouple demonstrated a wide thermopower tuned range compared to the recently reported *i*-TE thermocouples up to the present, as summarized in Fig. 4d. Moreover, after repeating the heating-cooling test in the air, the produced thermal voltage of the fabricated *i*-TEG demonstrated high repeatability after 50 cycles (Fig. 4f). Furthermore, to evaluate the generated power of the developed *i*-TE materials, an ionic thermoelectric capacitor (*i*-TEC) was built to convert ion charges originating from the heat to power an external load through 4 steps in one cycle as shown in Supplementary Fig. 18a. Briefly, a thermal voltage (*V*_1_) of a single PhNP film was generated at Δ*T* = 1.5 K. After connecting an external resistor, a pulse-like voltage (*V*_2_) of the external resistor was produced, which was caused by the accumulation of electrons and holes of the electrodes to balance *V*_1_. Then, the Δ*T* was removed and the external resistor was disconnected simultaneously, the *V*_1_ turned negative because the Na^+^ and TFSI^−^ recovered to the original status while the electrons and holes stayed at the two electrodes. The last step was to power the resistor by connecting to the *i*-TEC again (Supplementary Fig. 18b). The output energy was calculated according to E = ʃ*V*^2^/*Rdt*, where *V* and *R* are the voltage and the resistance of the external load, respectively^54^. The calculated power and energy of *i*-TEC was 2.08 µW cm^−2^ and 833.1 µJ cm^−2^ when a resistor with 1 kΩ was connected for the time duration 400 s of the last stage (Supplementary Fig. 18c, d), which is comparable to the recent works, such as 2.43 µW m^−2^ and 714 µJ m^−2^ of polyaniline (PANI)/poly(2-acrylamido-2-methyl-1-propane sulfonic acid) (PAAMPSA)/(phytic acid) (PA) with a resistor 6250 Ω^10^, 0.83 µW m^−2^ and 223.8 µJ m^−2^ of PVDF-HFP/1-ethyl-3-methylimidazolium dicyanamide (EMIM:DCA) with a resistor of 5 kΩ^8^.

### Light-induced thermal sensors

We further presented a self-powered and highly sensitive TS to detect light-induced heat by taking the unique advantages of the *i*-TE materials. The light-induced thermal sensor (LiTS) is fabricated with 4 pairs of PhNP and T-PhNP, where the *i*-TE legs are connected in series and suspended between the titanium nitride (TiN)^55^ absorber and the substrate (Fig. 4b). The TiN absorber was to generate heat under light illumination and the substrate was used as a heat sink. The LiTS could exhibit a huge advantage of high-temperature sensitivity over the commercial K-type thermocouple^56^ with a temperature sensitivity of 0.04 mV K^−1^. When the lamp is turned on, the voltage was suddenly generated by the LiTS and varied from −17.5 to −90 mV within the 50 s (Fig. 4c, g). Once the illumination is turned off, it can be observed the voltage dropped rapidly to the original status. After reducing the illumination power, the generated peak voltage decreased to −56 mV, which proved the sensing signal can be changed according to the variation of illuminance power correspondingly (Fig. 4g inset blue curve). Moreover, the sensor has been developed into a self-powered system that can automatically control the lamp without the need for an external amplifier circuit. As shown in Fig. 4h, once the light was on, the voltage was immediately generated of the system. When the voltage exceeded the upper limit, the system would automatically turn off the light. Then the temperature difference gradually decreased, and the voltage would drop accordingly. Until the lower limit was reached, the system would turn on the lights again and the voltage raised again. By changing the lighting power, the system can automatically control the on/off frequency of the lamp, where the greater lighting power has a faster response (Fig. 4i). Clearly, the developed self-powered system has the characteristics of high sensitivity, repeatability, and good consistency. Since the limiting factor for the speed of the *i*-TE LiTS is the bulk TE component, further miniaturization of the basic TE voltage generating unit will allow much shorter response times, which will be demonstrated in our future work. Overall, the *i*-TE based system provides higher voltage signals for the same thermal stimulus, which not only improves the resolution of the detector but also benefits from absolute large changes in voltage, especially for the heat-gated transistors, heat mapping^56,57^.

## Discussion

This work successfully developed all-solid-state PVDF-HFP/NaTFSI/PC with high thermopower (*S*_*i*_) of +20 mV K^−1^ attributed to the enhanced ions/polymer interactions and mass transport difference. Meanwhile, the *p*-*n* conversion in all-solid-state PhNP from +20 to −6 mV K^−1^ was achieved by incorporating TPFPB to impede the transportation of Na^+^ ions. Meanwhile, the mechanism of *p*-*n* conversion was studied in detail based on the analysis of Eastman entropy changes in all-solid-state PhNP *i*-TE polymers material at the atomic scale. In addition, this work also systematically investigated and optimized the TE performance of both *p*- and *n*-type materials with various compositions under different conditions. Moreover, an all-solid-state *i*-TE generator with 13 pairs of *p*-*n* legs generated a high voltage over 2.6 V (Δ*T* = 10 K). Besides, a self-powered TS demonstrated high sensitivity and good consistency. This work obtains high thermopower and realizes the *p*-*n* conversion in the all-solid-state *i*-TE polymer material simultaneously, which is beneficial for the development of next-generation high-performance flexible *i*-TE systems.

## Methods

### MD calculation of thermal conductivity and ionic diffusion of PhNP systems

All-atom MD simulations were performed to predict the thermal conductivity and ionic diffusion properties of the solid-state *i*-TE material PVDF-HFP/NaTFSI/PC. The initial structures of PVDF-HFP chains, NaTFSI, and PC molecules were generated using the freely available AVOGADRO software package^58^. These structures were then geometrically optimized via the use of Generalized Amber Force field (GAFF)^26^. The structures were considered to be geometrically optimized when the energy difference between two successive iterations dropped below a threshold value (10^−8^ kJ mol^−1^). The LigParGen web-based service^59^ is used to obtain the OPLS force-field parameters (Supplementary Fig. 19 and Supplementary Table 1) and partial atomic charges for the PVDF-HFP, NaTFSI, and PC molecules, which are placed together in a cubic simulation box with a dimension of 25 × 25 × 25 nm^3^ using PACKMOL^60^. We adjusted the number of PVDF-HFP, NaTFSI, and PC molecules so that the size of each system was kept consistent. Periodic boundary conditions in all three dimensions were implemented. The cut-off distance for long-range energy calculations was set to be 12 Å. The contribution of long-range interactions was calculated via the particle-particle-particle-mesh solver^61^. The Newton’s equations of motion were time-integrated with a time-step of 1 fs using Large-scale Atomic/Molecular Massively Parallel Simulation package^62^ developed by Sandia National Laboratories. The Visual Molecular Dynamics^63^ was used to visualize the trajectories generated during MD simulations. Each sample was equilibrated via the use of NPT simulations at 294 K and 1 atm over a period of 2 ns. Following this, a further 5 ns simulation was performed in the NVT ensemble. We recorded a trajectory of 1000 frames that were generated every 1 ps. The whole trajectory was then used for calculating the RDFs and mean square displacement curves (Supplementary Fig. 20). In thermal transport simulations, the heat source and heat sink were set as 320 and 280 K, respectively, using Langevin thermostats (Supplementary Fig. 21a). The system runs in the NVE ensemble for 1.5 ns to record heat flux and temperature gradient across the system (Supplementary Fig. 21b, c). The thermal conductivity of the PhNP-30, -50, and -80 samples was calculated based on Fourier law using non-equilibrium molecular dynamics simulation^64^.

## Supplementary information

Supplementary Information

## Data Availability

The authors declare that the main data supporting the findings of this study are available within the article and its Supplementary Information files. Extra data are available from the corresponding author upon reasonable request (maweigang@tsinghua.edu.cn).

## Code availability

The code of the entropy calculation is available at https://github.com/eastsheng/entropy.
