# Supplementary material for: Selectively tuning ionic thermopower in all-solid-state flexible polymer composites for thermal sensing
Source: Nat Commun. 2022 Jan 11;13:221. doi: 10.1038/s41467-021-27885-2 (PMC8752756; doi:10.1038/s41467-021-27885-2)
Supplement: Supplementary file 1 — Supplementary Information [file 41467_2021_27885_MOESM1_ESM.pdf]

Supplementary Information

Selectively Tuning Ionic Thermopower in All-Solid-State Flexible Polymer Composites for Thermal Sensing

Cheng Chi[1ǂ], Meng An[1,2ǂ], Xin Qi[1], Yang Li[3], Ruihan Zhang[4], Gongze Liu[3], Chongjia Lin[3], He Huang[3], Hao Dang[1], Baris Demir[5], Yan Wang[4], Weigang Ma[1*], Baoling Huang[3*] and Xing Zhang[1]

[1]Key Laboratory for Thermal Science and Power Engineering of Ministry of Education, Department of Engineering Mechanics, Tsinghua University, Beijing, 100084, China

[2]College of Mechanical & Electrical Engineering, Shaanxi University of Science and Technology, Xi'an, 710021, China

[3]Department of Mechanical and Aerospace Engineering, The Hong Kong University of Science and Technology, Clear Water Bay, Hong Kong SAR, China

[4]Department of Mechanical Engineering, Worcester Polytechnic Institute, 100 Institute Road, Worcester, MA 01609, USA

[5]Centre for Theoretical and Computational Molecular Science, The Australian Institute for Bioengineering and Nanotechnology, The University of Queensland, Brisbane, Queensland 4072, Australia

***Corresponding author:** W. G. Ma, email: maweigang@tsinghua.edu.cn; B. L. Huang, email: mebhuang@ust.hk

ǂThe authors contribute equally.

Inventory of Supplementary Information:

Supplementary Figures

Supplementary Fig. 1 Images of the flexible all-solid-state *i*-TE materials

Supplementary Fig. 2 Home-made ionic Seebeck coefficient measurement setup

Supplementary Fig. 3 The plot of ΔV_i-ΔT curves of each *p*-type PhNPs

Supplementary Fig. 4 Electrochemical impedance spectra results

Supplementary Fig. 5 The surface morphology of *p*-type PhNPs

Supplementary Fig. 6 The measurement setup of the hot disk method

Supplementary Fig. 7 The thermoelectric property of PhNP with various amounts of NaTFSI

Supplementary Fig. 8 The Raman spectra of each PhNPs

Supplementary Fig. 9 The schematic illustration of ion clusters and transportation

Supplementary Fig. 10 The surface morphology of *n*-type T-PhNP

Supplementary Fig. 11 The measured thermopower of each *n*-type T-PhNP.

Supplementary Fig. 12 The XPS spectra of fabricated *i*-TE materials

Supplementary Fig. 13 The calculated entropy change of PhNP and T-PhNP

Supplementary Fig. 14 The ionic thermoelectric properties of *n*-type T-PhNP

Supplementary Fig. 15 The humidity effect on the thermopower

Supplementary Fig. 16 The images of the *i*-TE materials after the PVD process

Supplementary Fig. 17 The ΔV_i-ΔT curves of the fabricated *i*-TEG

Supplementary Fig. 18 The performance of the ionic thermoelectric capacitor

Supplementary Fig. 19 The atom type force field in the molecular dynamic simulations

Supplementary Fig. 20 The mean-squared displacement of anion and cation

Supplementary Fig. 21 The calculations of the thermal conductivity

Supplementary Table 1. The parameters in potential functions

Supplementary Methods

Supplementary Discussion

Supplementary References

Supplementary Figures

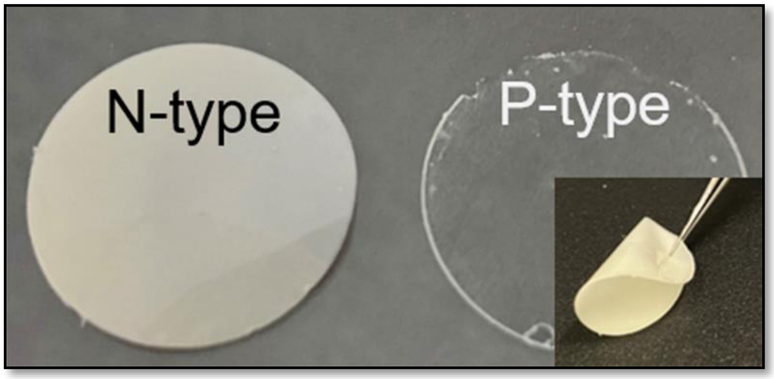

Supplementary Fig. 1. **Images of the flexible all-solid-state *i*-TE materials**. The *n*-type T-PhNP is white while the *p*-type PhNP is transparent.

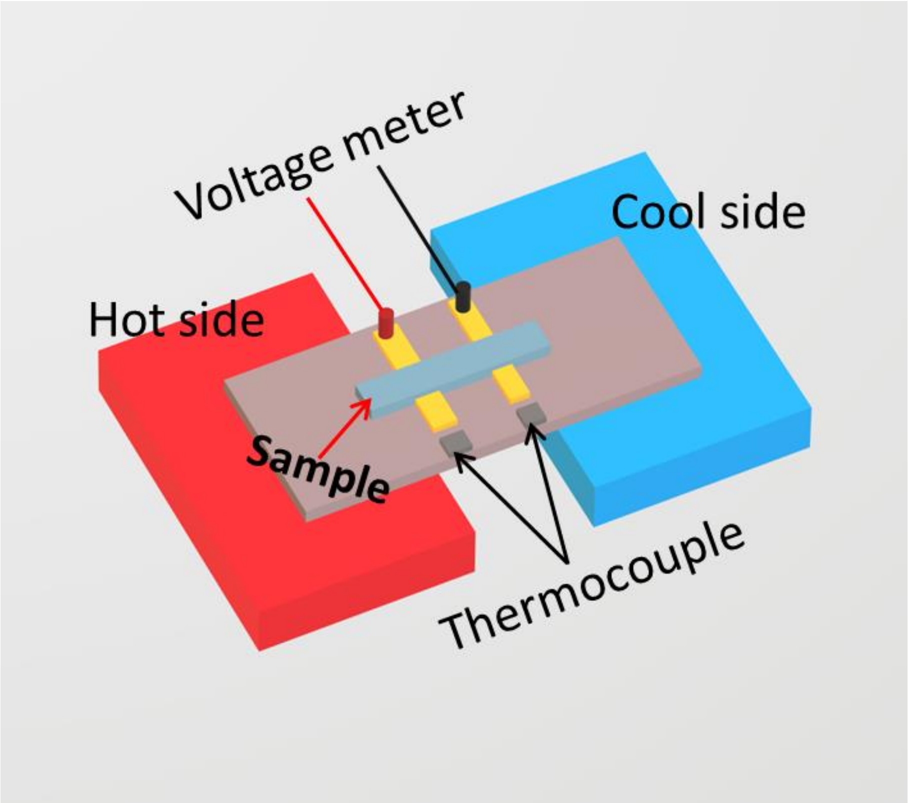

Supplementary Fig. 2. **Home-made ionic Seebeck coefficient measurement setup**. The red and blue regions denote the heat source and heat sink, respectively. The temperature and voltage are recorded by K-type thermocouple and voltage meter, respectively.

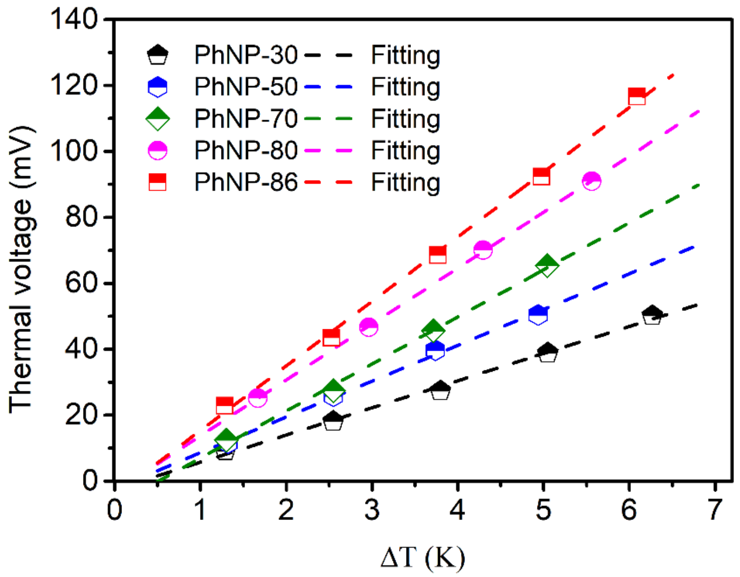

Supplementary Fig. 3. **The plot of ΔV_i-ΔT curves of each *p*-type PhNPs**. The weight ratios of NaTFSI/PC to overall composite range from 30wt.% to 86wt.% and the corresponding dash line is obtained by linearly fitting.

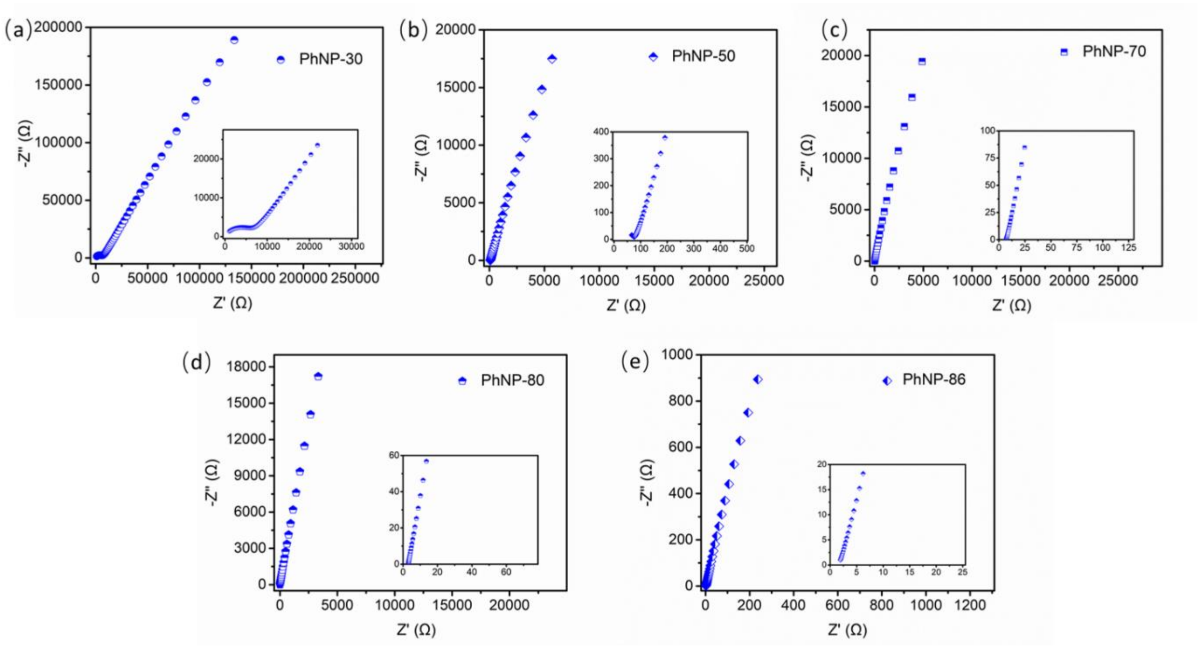

Supplementary Fig. 4. **Electrochemical impedance spectra results**. Electrochemical impedance spectra curves of (a) PhNP-30, (b) PhNP-50, (c) PhNP-70, (d) PhNP-80 and (e) PhNP-86. Insert: the magnification part at higher frequency.

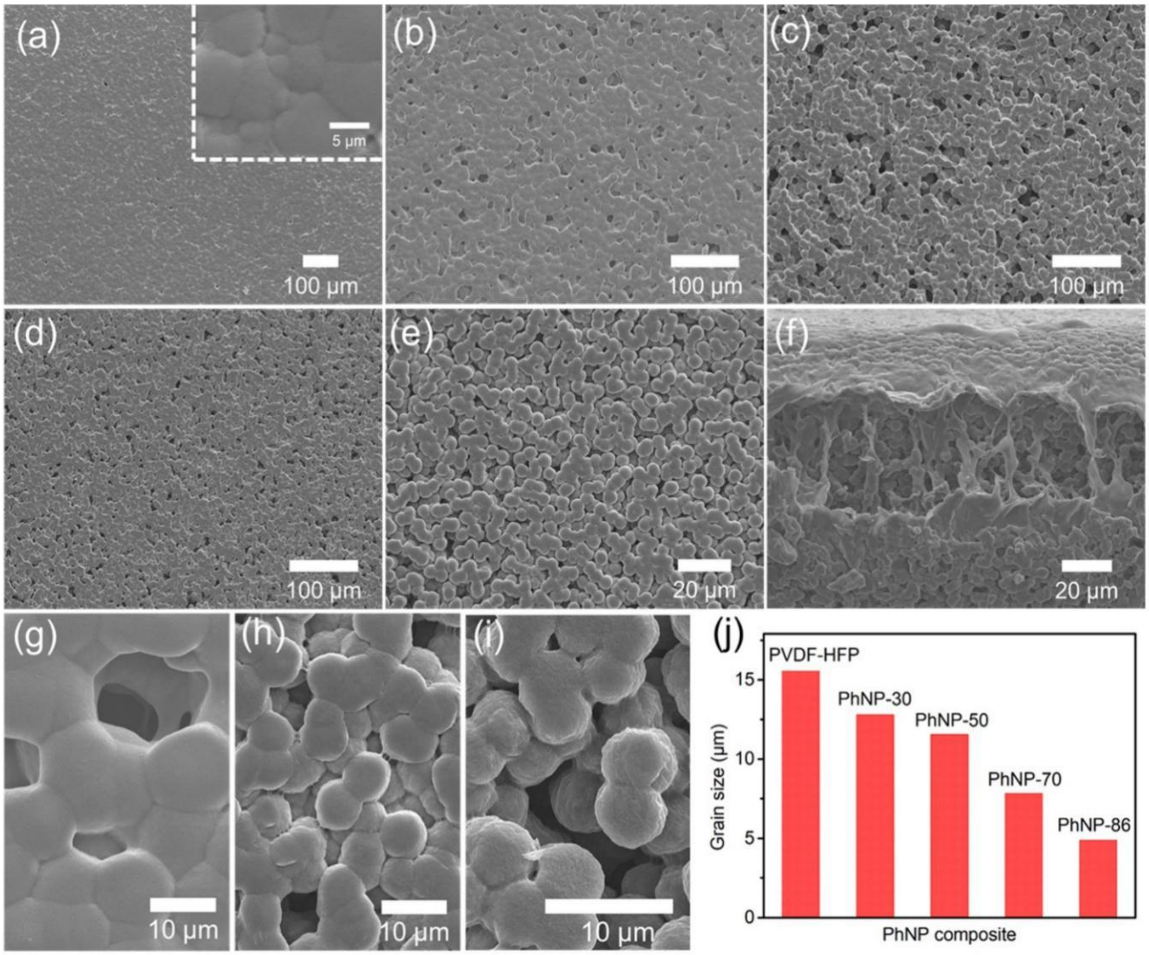

Supplementary Fig. 5. **The surface morphology of *p*-type PhNPs**. SEM images of top view of (a) pure PVDF-HFP (The inset: magnification part), (b) PhNP-30, (c) PhNP-50, (d) PhNP-70, (e) PhNP-86 and (f) cross-section view of PhNP-86. The magnification part of (g) PhNP-30, (h) PhNP-70 and (i) PhNP-86 with sphere-shape-like grains. (j) The statistic of the average grain size of each PhNPs.

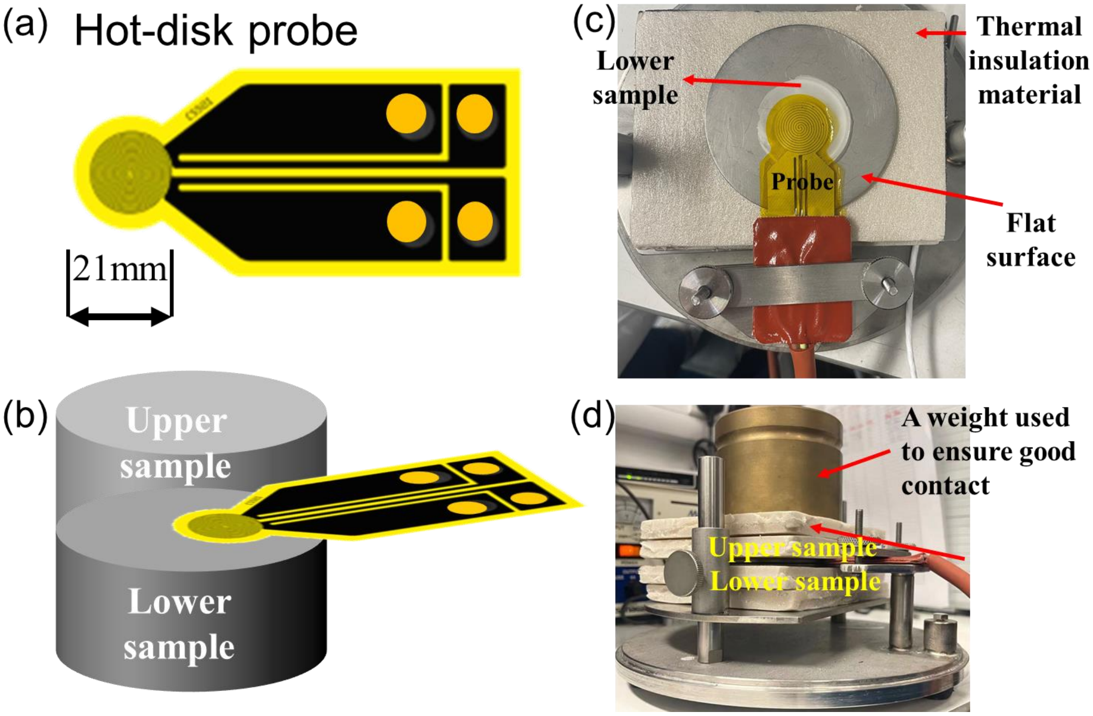

Supplementary Fig. 6. **The measurement setup of the hot disk method**. (a) The hot disk probe and (b) the diagram of the test structure where the probe was sandwiched with two same samples. (c) and (d) The experimental measurement setup of the TPS 2500S model.

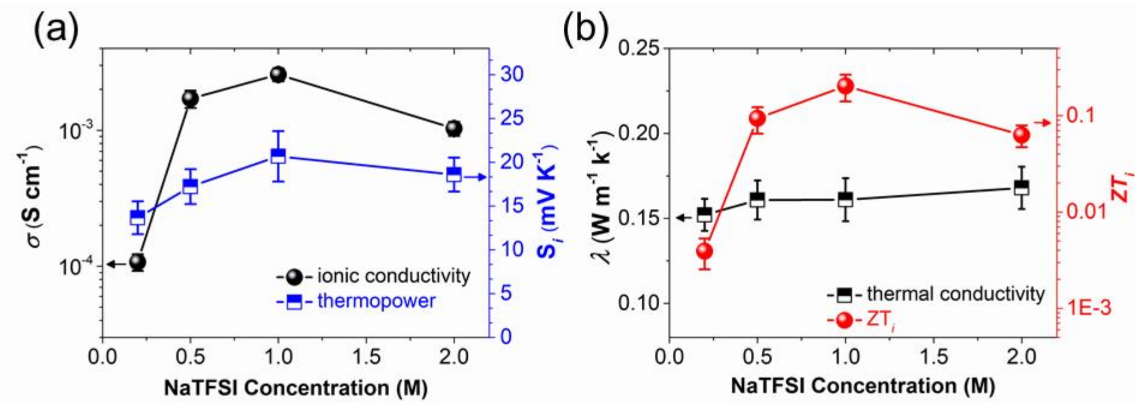

Supplementary Fig. 7. **The thermoelectric property of PhNP with various amounts of NaTFSI**. The ionic thermoelectric properties (a) the ionic conductivity, thermopower, and (b) thermal conductivity, figure of merit ZT_i of PhNP-86 samples with different concentrations of NaTFSI from 0.2 M to 2 M.

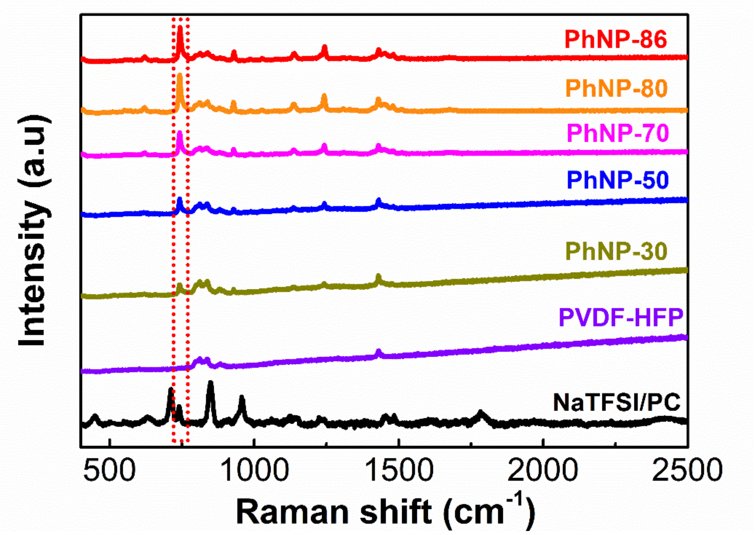

Supplementary Fig. 8. **The Raman spectra of each PhNPs**. The Raman spectra were performed with a range of 400-2500 cm^{-1} of each PhNPs, pure PVDF-HFP, and NaTFSI/PC. The weight ratios of NaTFSI/PC to overall composite range from 30wt.% to 86wt.%.

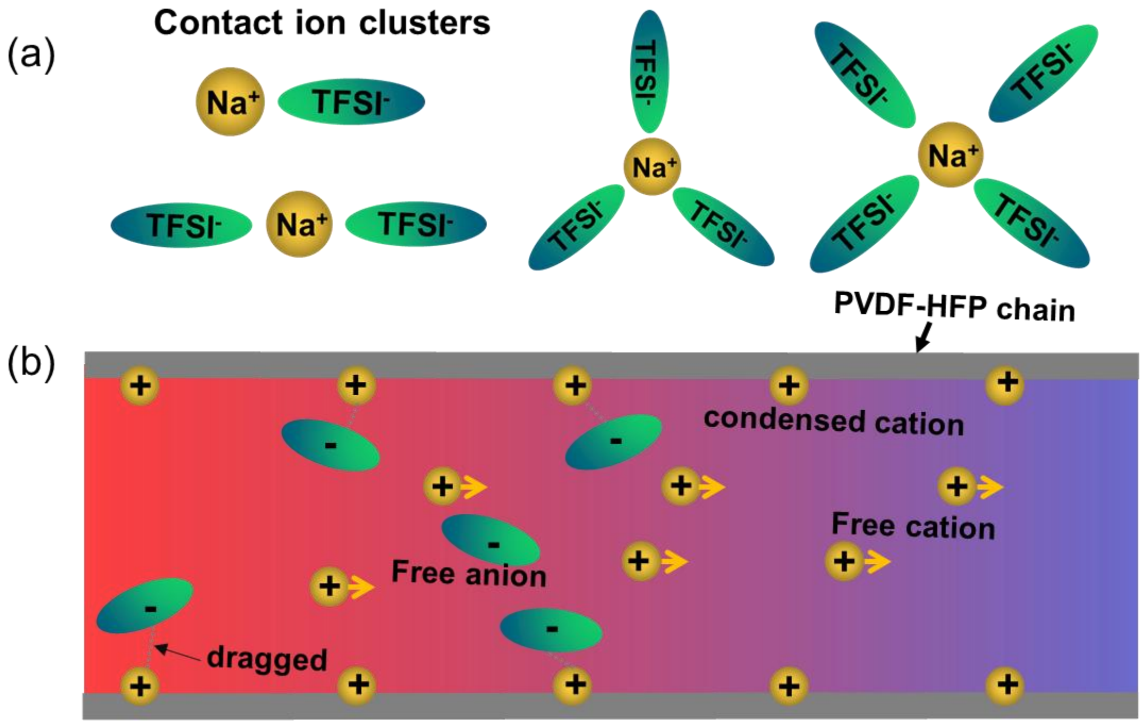

Supplementary Fig. 9. **The schematic illustration of ion clusters and transportation**. (a) The illustration of formed contact ion complex and (b) The schematic of the enhanced Na^+ ion mobility along negatively charged polymer chains.

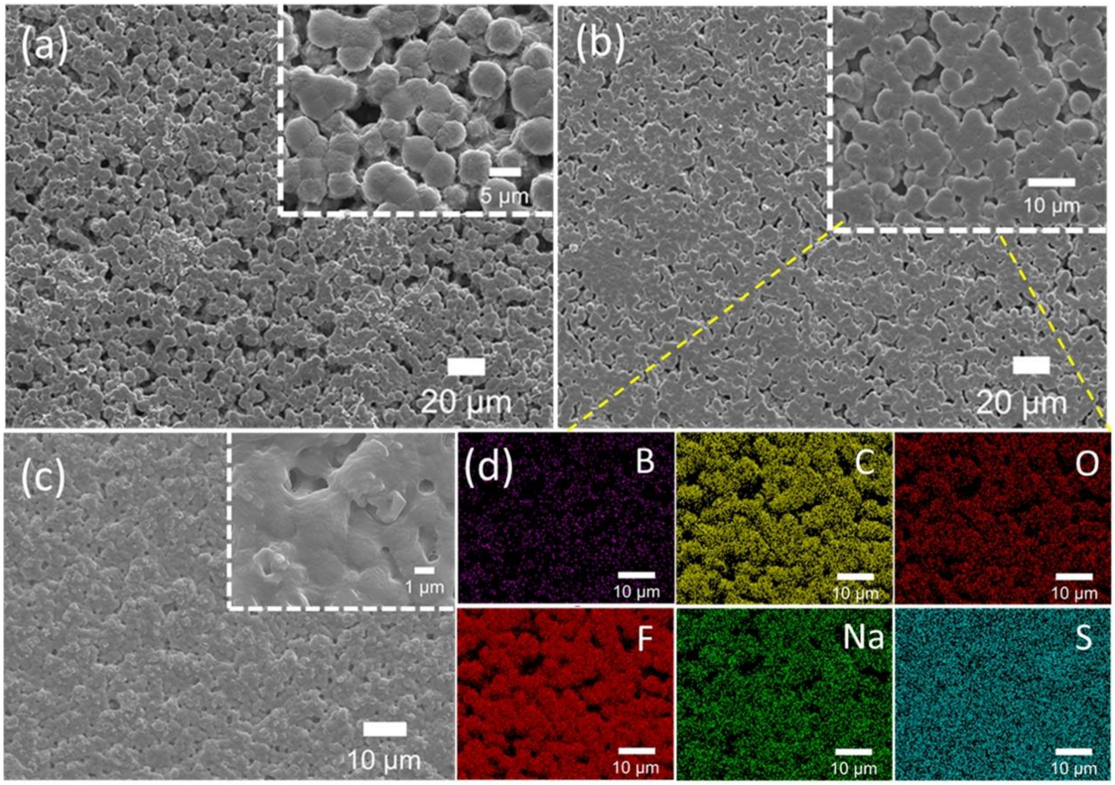

Supplementary Fig. 10. **The surface morphology of *n*-type T-PhNP**. The SEM images of the fabricated all-solid-state (a) T-PhNP-0.1M, (b) T-PhNP-0.5M (c) T-PhNP-1.5M with the inset of the magnification part, and (d) the EDS mapping and the element distribution of T-PhNP-0.5M.

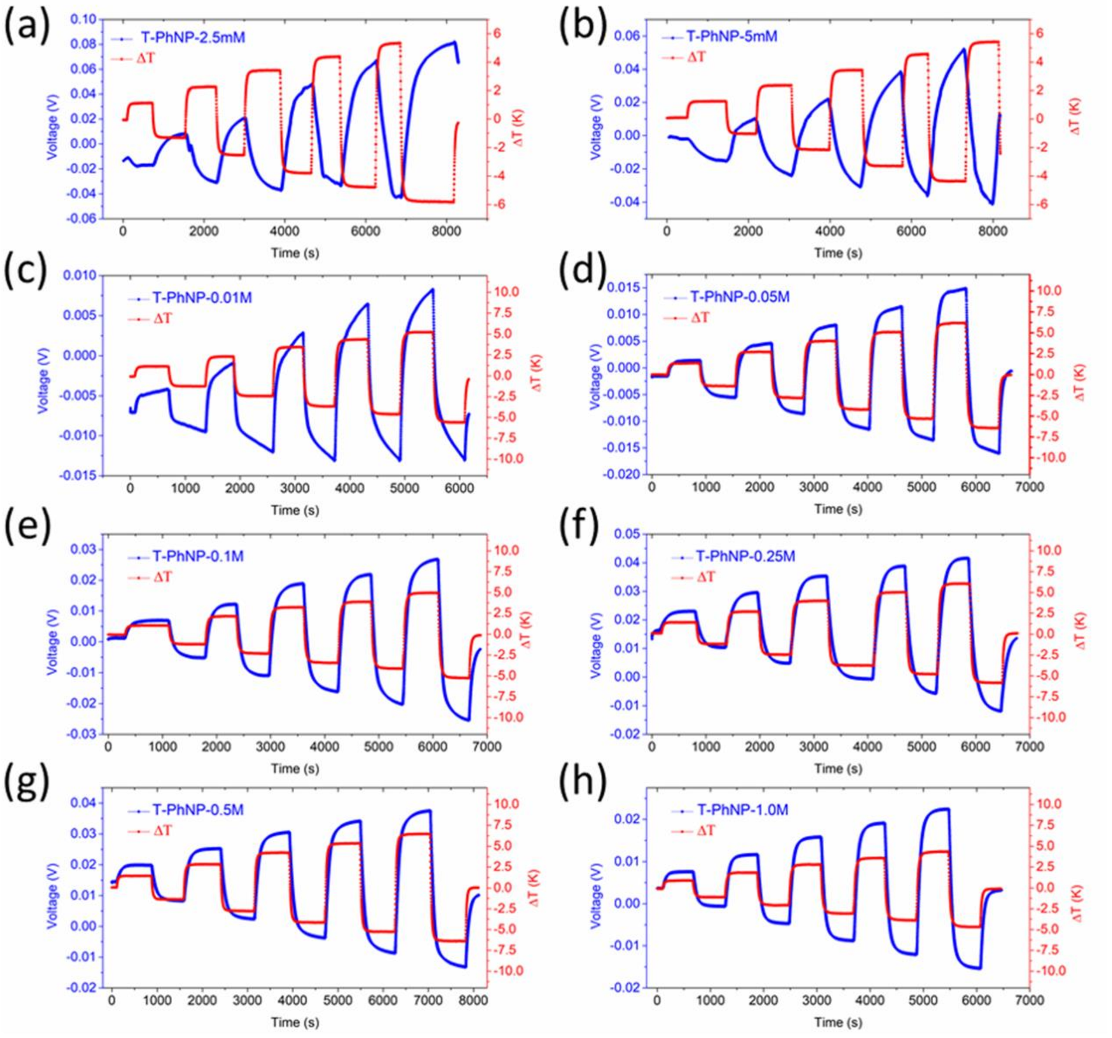

Supplementary Fig. 11. **The measured thermopower of each *n*-type T-PhNP**. The ΔV_i-ΔT curves of each T-PhNPs with different concentrations with (a) 2.5 mM, (b) 5 mM, (c), 0.01 M, (d) 0.05 M, (e) 0.1M, (f) 0.25 M, (g) 0.5 M and (h) 1.0 M of TPFPB.

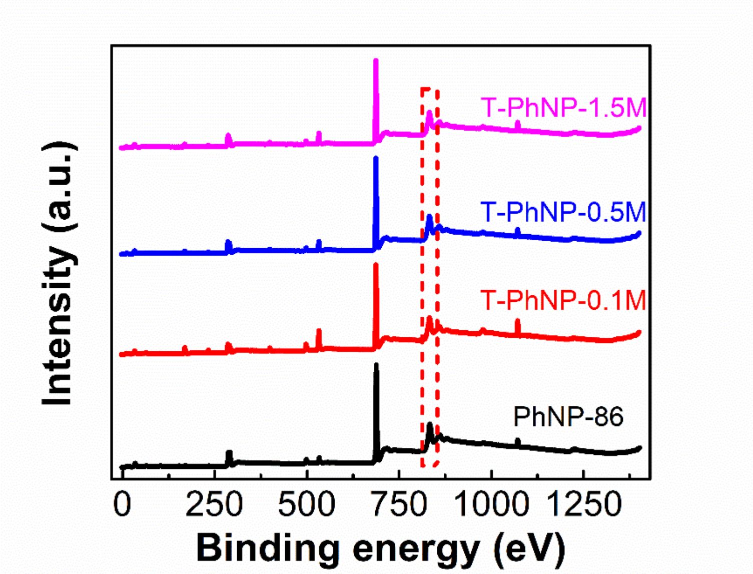

Supplementary Fig. 12. **The XPS spectra of fabricated *i*-TE materials**. The XPS analysis was conducted to study the binding energy of *p*-type PhNP-86 and T-PhNPs with different amounts of TPFPB.

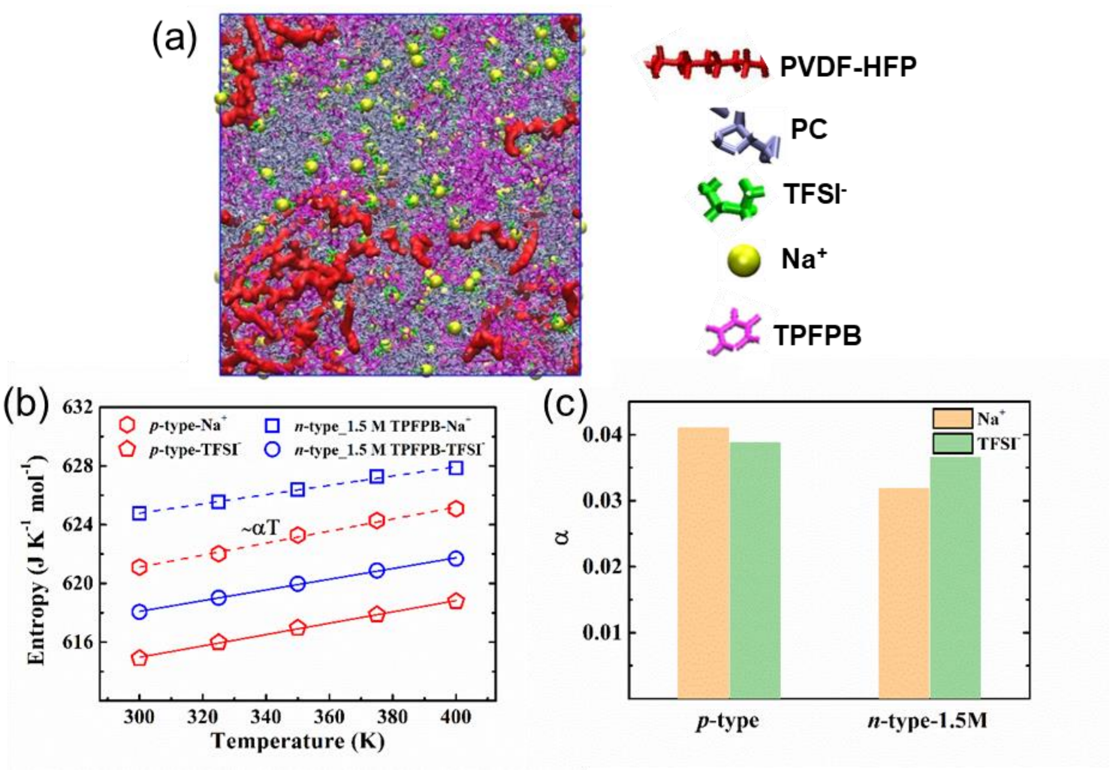

Supplementary Fig. 13. **The calculated entropy change of PhNP and T-PhNP**. (a) The snapshots of *n*-type T-PhNP-1.5M samples in the MD simulation model. (b) The entropy of cation and anion in PhNP-80 and T-PhNP-1.5M system with the temperature from 300 to 400 K. (c) The linear fitting coefficient of cation and anion in *p*-type PhNP-80 and *n*-type T-PhNP-1.5M.

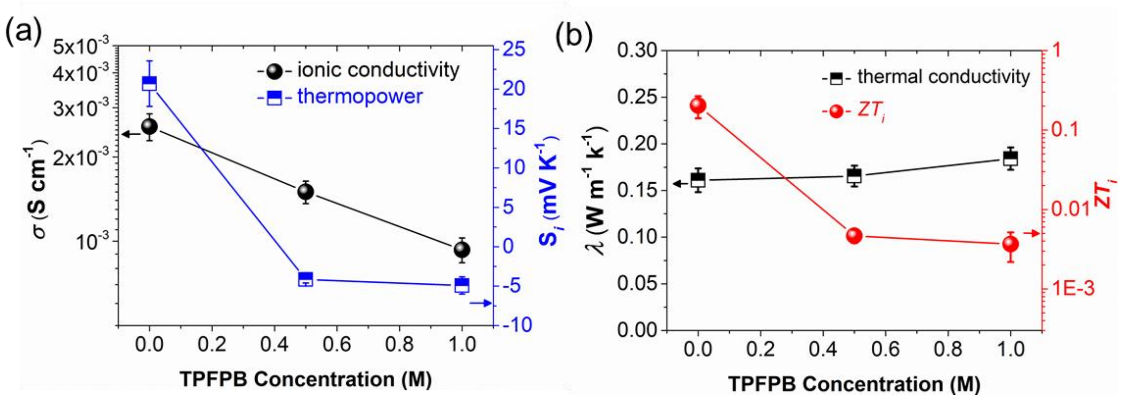

Supplementary Fig. 14. **The ionic thermoelectric properties of *n*-type T-PhNP**. (a) The ionic conductivity, thermopower, and (b) thermal conductivity and the figure of merit ZT_i of each T-PhNP with different concentrations of TPFPB.

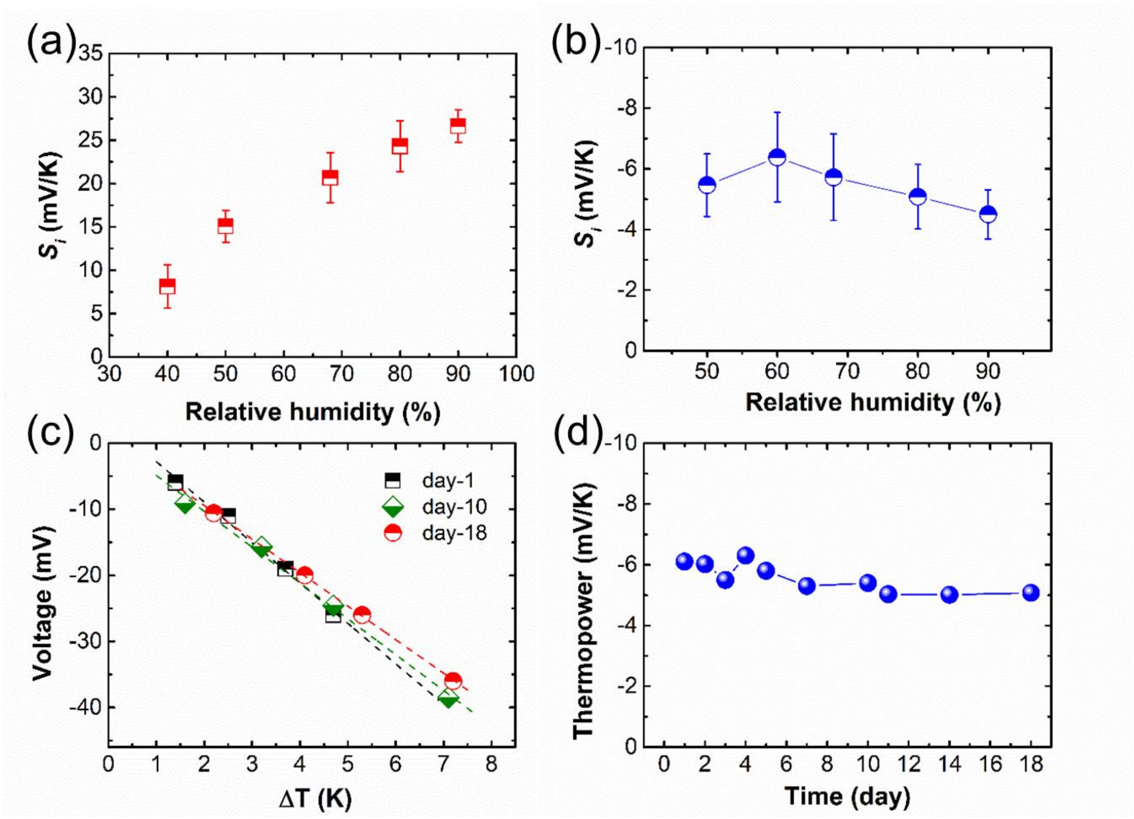

Supplementary Fig. 15. **The humidity effect on the thermopower**. (a) The thermopower of the *p*-type PhNP-86 sample under various humidity conditions from 40% RH to 90% RH. (b)The thermopower of the *n*-type T-PhNP-1.0M sample under various humidity conditions from 50% RH to 90% RH. (c) The plot of the ΔV vs ΔT fitting curves and (d) the stability test of *n*-type T-PhNP-1.0M sample at 68% RH over 18 days.

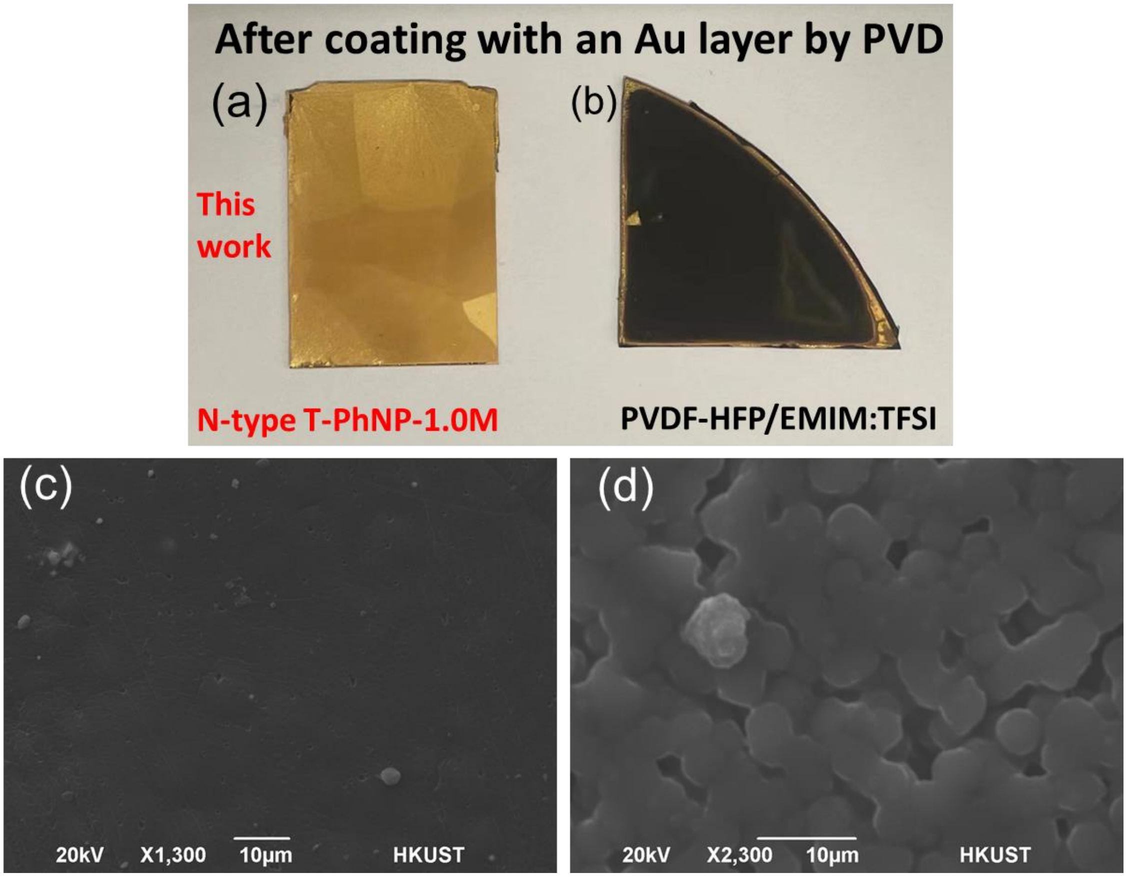

Supplementary Fig. 16. **The images of the *i*-TE materials after the PVD process**. The digital photos of (a) T-PhNP developed in this work and (b) PVDF-HFP/EMIM:TFSI after coating with an Au layer by the same PVD process. The SEM images of the (c) T-PhNP and (d) PVDF-HFP/EMIM:TFSI composites.

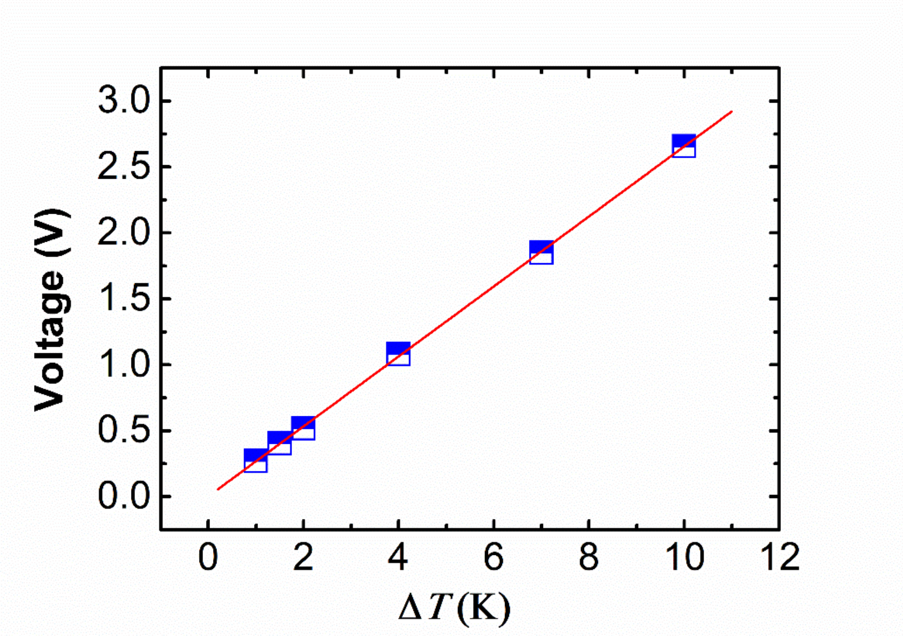

Supplementary Fig. 17. **The ΔV_i-ΔT curves of the fabricated *i*-TEG**. The blue squares are the measured voltage results as a function of temperature differences. The red line is obtained by linearly fitting.

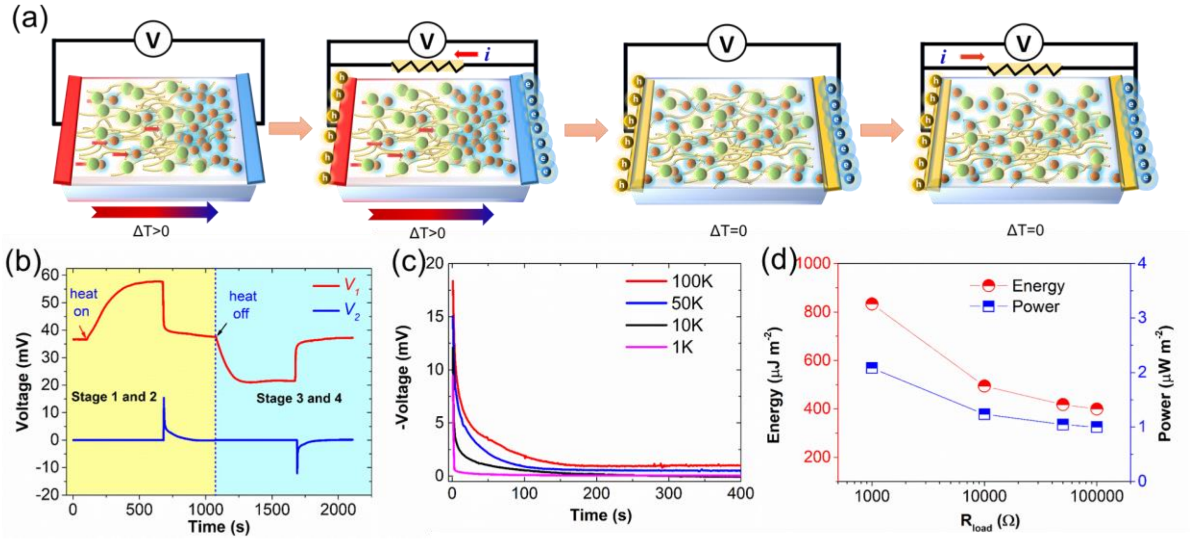

Supplementary Fig. 18. **The performance of the ionic thermoelectric capacitor**. (a) The 4 steps working principle in one thermal cycle of *i*-TEC. (b) The voltage profiles of the *i*-TEC device using a 10 kΩ resistor as external load (red line) and of the external load (blue line). (c) Voltage (V_2) decay curves of the external loading with different resistances connected to the i-TEC and (d) the total discharging energy and power versus the resistance of the external loading.

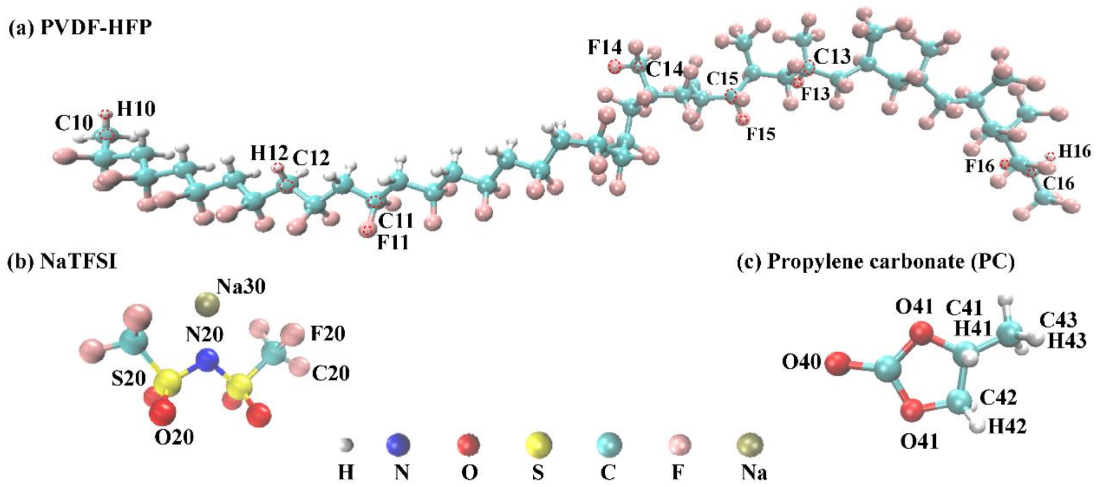

Supplementary Fig. 19. **The atom type force field in the molecular dynamic simulations**. (a) The atom force field type of PVDF-HFP. (b) The atom force field type of NaTFSI. (c) The atom force field type of propylene carbonate.

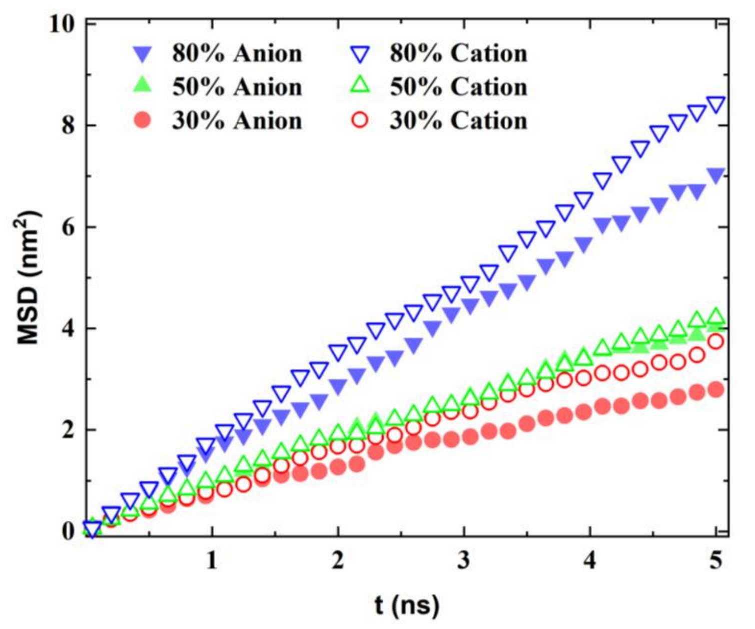

Supplementary Fig. 20. **The mean-squared displacement of anion and cation**. The solid and hollow symbols are the MSD of anion and cation for different PC/NaTFSI of three PhNP-30, -50 and -80 systems, respectively.

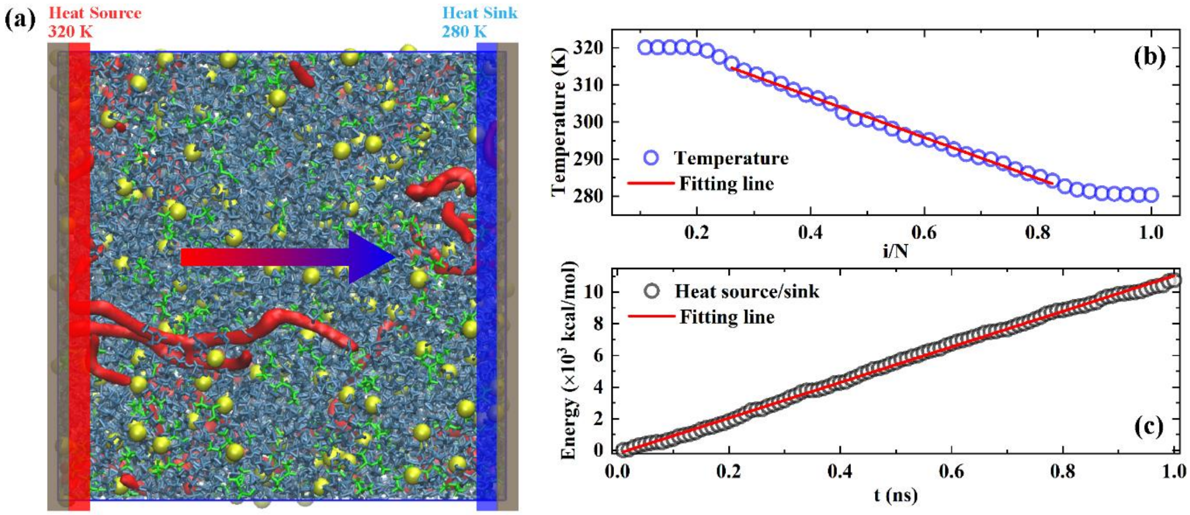

Supplementary Fig. 21. **The calculations of the thermal conductivity**. (a) Simulation setup for thermal conductivity calculations in the non-equilibrium molecular dynamics simulations, where heat flux across the system from the heat source to heat sink with the fixed regions at the two ends of the system. (b) The steady-state temperature profile of PVDFHFP/NaTFSI/PC system was used for thermal conductivity calculations. (c) The cumulative energy input and output in the thermostated regions from the NEMD simulations.

Supplementary Table 1. The parameters in potential functions.

Bond Parameters

Bond	K_r (Kcal/(mol·Å^2))	r_{eq} (Å)
C10-C11	268.0000	1.5290
C10-H10	340.0000	1.0900
C11-C11	268.0000	1.5290
C11-C12	268.0000	1.5290
C11-C13	268.0000	1.5290
C11-F11	367.0000	1.3600
C12-H12	340.0000	1.0900
C13-C14	268.0000	1.5290
C13-C15	268.0000	1.5290
C13-F13	367.0000	1.3600
C14-C16	268.0000	1.5290
C14-F14	367.0000	1.3600
C15-C16	268.0000	1.5290
C15-F15	367.0000	1.3600
C16-F16	367.0000	1.3600
C16-H16	340.0000	1.0900
C20-F20	441.92	1.323
C20-S20	233.03	1.818
C40-O40	570.00	1.2290
C40-O41	214.00	1.3270

C41-C42	268.00	1.5290
C41-C43	268.00	1.5290
C41-H41	340.00	1.0900
C41-O41	320.00	1.4100
C42-H42	340.00	1.0900
C42-O41	320.00	1.4100
C43-H43	340.00	1.0900
N20-S20	374.88	1.570
O20-S20	637.07	1.437

Angle Parameters

**Bond**	**K_θ (Kcal/(mol·radian^2))**	**θ_{eq} (degree)**
C10-C11-C12	58.350	112.700
C10-C11-F11	50.000	109.500
C11-C10-H10	37.500	110.700
C11-C11-C12	58.350	112.700
C11-C11-C13	58.350	112.700
C11-C11-F11	50.000	109.500
C11-C12-C11	58.350	112.700
C11-C12-H12	37.500	110.700
C11-C13-C14	58.350	112.700
C11-C13-C15	58.350	112.700
C11-C13-F13	50.000	109.500
C12-C11-C12	58.350	112.700

C12-C11-F11	50.000	109.500
C13-C11-F11	50.000	109.500
C13-C14-F14	50.000	109.500
C13-C15-C13	58.350	112.700
C13-C15-C16	58.350	112.700
C13-C15-F15	50.000	109.500
C14-C13-C15	58.350	112.700
C14-C13-F13	50.000	109.500
C14-C16-C15	58.350	112.700
C14-C16-F16	50.000	109.500
C14-C16-H16	37.500	110.700
C15-C13-C15	58.350	112.700
C15-C13-F13	50.000	109.500
C15-C16-F16	50.000	109.500
C15-C16-H16	37.500	110.700
C16-C14-F14	50.000	109.500
C16-C15-F15	50.000	109.500
C20-S20-N20	91.30	103.5
C20-S20-O20	103.97	102.6
C40-O41-C41	83.00	116.90
C40-O41-C42	83.00	116.90
C41-C42-H42	37.50	110.70
C41-C42-O41	50.00	109.50

C41-C43-H43	37.50	110.70
C42-C41-C43	58.35	112.70
C42-C41-H41	37.50	110.70
C42-C41-O41	50.00	109.50
C43-C41-H41	37.50	110.70
C43-C41-O41	50.00	109.50
F11-C11-F11	77.000	109.100
F14-C14-F14	77.000	109.100
F15-C15-F15	77.000	109.100
F16-C16-H16	40.000	107.000
F20-C20-F20	93.33	107.1
F20-C20-S20	82.93	111.7
H10-C10-H10	33.000	107.800
H12-C12-H12	33.000	107.800
H41-C41-O41	35.00	109.50
H42-C42-H42	33.00	107.80
H42-C42-O41	35.00	109.50
H43-C43-H43	33.00	107.80
N20-S20-O20	94.29	113.60
O20-S20-O20	115.80	118.5
O40-C40-O41	83.00	123.40
O41-C40-O41	81.00	111.40
S20-N20-S20	80.19	125.6

VDW Parameter

Atom type	ε(Kcal/mol)	σ (Å)
C10	0.0660	3.5000
C11	0.0660	3.5000
C12	0.0660	3.5000
C13	0.0660	3.5000
C14	0.0660	3.5000
C15	0.0660	3.5000
C16	0.0660	3.5000
C20	0.0660	3.5000
C40	0.1050	3.7500
C41	0.0660	3.5000
C42	0.0660	3.5000
C43	0.0660	3.5000
F11	0.0600	2.9000
F13	0.0600	2.9000
F14	0.0600	2.9000
F15	0.0600	2.9000
F16	0.0600	2.9000
F20	0.0530	2.9500
H10	0.0300	2.5000
H12	0.0300	2.5000
H16	0.0300	2.5000

H41	0.0150	2.4200
H42	0.0150	2.4200
H43	0.0150	2.4200
N20	0.1700	3.2500
NA30	0.0005	4.0700
O20	0.2100	2.9600
O40	0.2100	2.9600
O41	0.1700	3.0000
S20	0.2500	3.5500

Supplementary methods

Materials

Polyvinylidenefluoride-hexafluoropropylene pellet (PVDF-HFP, average M_w, 455,000 g mol^{-1}), sodium bis(trifluoromethylsulfonyl)imide (NaTFSI), propylene carbonate (PC, anhydrous, 99.7%), and *N*-Methyl-2-pyrrolidone (NMP, HPLC, ≥99%) were purchased from Sigma-Aldrich. Tris(pentafluorophenyl)borane (TPFPB, min. 97%) was purchased from Strem Chemicals, Inc. All the materials were stored in the glovebox without any additional treatment.

Preparation of PhNPs and T-PhNPs

PVDF-HFP pellets were firstly dried in an oven at 100°C for 24 h and were dissolved in NMP at a concentration of 0.1 g mL^{-1} with rapid magnetic stirring for about 8 h at 60°C. Next, 1M NaTFSI/PC solution with different mass ratios from 30 to 86 wt. % was added to the PVDF-HFP solution to form a homogeneous mixture in an argon atmosphere. The resulting mixture was then cast on a glass petri-dish and dried in a vacuum oven (10^{-3} Torr) at 60°C for over 1~10 h to obtain the free-standing PhNP films. For T-PhNPs, different amount of TPFPB was added to PhNP-86 mixtures at 60°C and then followed the same procedures to obtain free-standing solid-state T-PhNP films. All the films were stored in a glove box (O_2 < 0.1 ppm, and H_2O < 0.1 ppm) for future use. Here, the weight ratio means the liquid NaTFSI/PC is to the overall weight of the PVDF-HFP/NaTFSI/PC composites as illustrated by *Eq*. (1)

$$W_{(\mathrm{NaTFSI/PC})}=m_{(\mathrm{NaTFSI/PC})}/(m_{(\mathrm{NaTFSI/PC})}+m_{PVDF\text{-}HFP}) \qquad (1)$$

where $m_{(\mathrm{NaTFSI/PC})}$ and $m_{PVDF\text{-}HFP}$ represented the mass of NaTFSI/PC and PVDF-HFP, respectively.

Fabrication of i-TE Device

The as-fabricated *p*-type PhNP and *n*-type T-PhNP films were cut into identical thin-rectangular shapes with a dimension of 6×1.5 mm^2. The 13 pairs of the developed thermocouple films were carefully transferred to the corresponding electrodes. Next was to make sure the *p*-type and *n*-type films were electrically connected in series with the bridge gap between the hot side and cool side larger than 2.0 mm. Then, the in-plane all-solid-state *i*-TE generator module prototype can be ready for further test.

Characterization

A scanning electron microscope (SEM, JEOL-7100F) was used to characterize the morphology of the materials and element distribution. The samples were sputter-coated with approximately 10 nm of gold before the analysis. FTIR characterization was conducted using Bruker (Vertex 70 Hyperion 1000) within the range of 400-950 cm^{-1} and the resolution of the spectra was 4 cm^{-1}. Raman characterization was conducted using InVia (Renishaw) within the range of 100-2500 cm^{-1} and a laser source of 633 nm. X-ray photoelectron spectroscopy measurement was conducted using PHI 5600.

Ionic conductivity measurement

The ionic conductivity of the *i*-TE materials PVDF-HFP/NaTFSI/PC was determined by the electrochemical impedance spectroscopy (EIS) measurement in a frequency range from 100 kHz to 0.1 Hz using two stainless steel (SS) block electrodes (SS//CPE//SS). The ion conductivity was calculated according to *Eq*. 2:

$$\sigma = \frac{l}{R_b A} \tag{2}$$

where l and A are the thickness and contact area of the materials, respectively. The bulk electrolyte resistance value R_b was determined from the intercept of the semi-circular curve with the real axis at the low-frequency region. This method was widely used in the literature and calibrated in our previous work.

Thermal conductivity measurement

The thermal conductivity of the *i*-TE materials PVDF-HFP/NaTFSI/PC was determined by Hot disk thermal constant analyzer (TPS 2500S, Sweden) with a Kapton-insulated sensor 7854 at room temperature as shown in Supplementary Fig. 5. The transient plane source (TPS) method utilizes the Kapton-insulated sensors as the temperature sensor and heat source. The diameter of PVDF-HFP films should be larger than 2.1 centimeters and thickness should be limited from 10 to 500 microns.

All the measurements including thermopower, ionic conductivity and thermal conductivity are performed using at least 5 separate samples and the error bars are calculated using standard derivation.

Supplementary discussion

The potential energy of the systems U in our work was evaluated as a sum of individual energies (*Eq*. 3) for harmonic bond stretching and angle bending terms, a cosine series for torsional energetics, Coulomb, and 12-6 Lennard-Jones terms for the nonbonded interactions (*Eq.* 3-7).

$$U = E_{bonds} + E_{angles} + E_{torsion} + E_{nonbond} \tag{3}$$

$$E_{bonds} = \sum_{bonds} k_b (r - r_{eq})^2 \quad (4)$$

$$E_{angles} = \sum_{angles} k_\theta (\theta - \theta_{eq})^2 \quad (5)$$

$$E_{torsion} = \sum_i \left[\frac{1}{2}V_{1,i}(1 + cos\phi) + \frac{1}{2}V_{2,i}(1 - cos2\phi) + \frac{1}{2}V_{3,i}(1 + cos3\phi) + \frac{1}{2}V_{4,i}(1 - cos4\phi)\right] \quad (6)$$

$$E_{nonbond} = \sum_i \sum_{j>i} \left\{\frac{q_i q_j e^2}{r_{ij}} + 4\varepsilon_{ij}\left[\left(\frac{\sigma_{ij}}{r_{ij}}\right)^{12} - \left(\frac{\sigma_{ij}}{r_{ij}}\right)^6\right]\right\} \quad (7)$$

where the parameters are the force constant *k*, the r_{eq}, θ_{eq}, equilibrium bond and angle values, Fourier coefficient *V*, partial atomic charges, q, and Lennard-Jones radii and well-depths, σ and ε. The σ and ε used in *Eq. 6* for an interaction of atom *i* and atom *j* is $\sigma_{ij} = \sigma_i + \sigma_j$ and $\varepsilon_{ij} = \sqrt{\varepsilon_i \cdot \varepsilon_j}$, respectively. In our systems, all the atomic types in empirical potential are shown in Supplementary Fig. 19.

Preparation of ionic thermoelectric material samples in MD simulations

The initial structures of PVDF-HFP chains, NaTFSI and PC molecules were generated using the freely available AVOGADRO software package[1]. These structures were then geometrically optimized via the use of the Generalized Amber Force field (GAFF)[2]. The structures were considered to be optimized when the energy difference between two successive interactions dropped below a threshold value (10^{-8} kJmol^{-1}). We used LigParGen web-based service[3] that can provide the OPLS force-field parameters and partial atomic charges for the PVDF-HFP, NaTFSI, and PC molecules. We generated a solid-state ionic thermoelectric (*i*-TE) material sample by placing PVDF-HFP, NaTFSI and PC molecules together in a cubic simulation box with a dimension of 25.0 nm using PACKMOL[4]. We adjusted the number of PVDF-HFP, NaTFSI, and PC molecules so that the size of each system was similar. Each sample was equilibrated via the

use of NPT-MD simulations at 294 K and 1 atm over a period of 1 ns. Each ionic thermoelectric material sample was generated separately in the simulation box where all PVDF and HFP monomers, NaTFSI, and PC molecules were present. Then, the thermoelectric material samples were equilibrated via the use of NPT-MD simulations at 294 K and 1 atm over a period of 1 ns. Following this, a further 1 ns simulation was performed in the NVT-MD. We tracked a trajectory of 1000 frames that were generated every 1 ps. The whole trajectory was then used for calculating the radial distribution function and mean-squared displacement. The Newton's equations of motion were time-integrated with a time-step of 1 fs implemented by the Large-scale Atomic/Molecular Massively Parallel Simulation (LAMMPS) package[5] developed by Sandia National Laboratories. The Visual Molecular Dynamics (VMD)[6] was used to visualize the trajectories generated during simulations.

Calculation of ion diffusion and thermal conductivity

All-atom molecular dynamics (MD) simulations were used to predict thermal conductivity and ionic transport properties of the solid-state ionic thermoelectric (*i*-TE) material PVDF-HFP/NaTFSI/PC (PhNP). Periodic boundary conditions in all three dimensions were implemented. The Nosé-Hoover thermostat and barostat were implemented in all the simulations to control the temperature and pressure, respectively. The cut-off distance for long-range energy calculations was set to be 12 Å. The contribution of long-range interactions was calculated via the particle-particle-particle-mesh (PPPM) solver[7]. We predict the diffusion coefficient using results from NVT-MD simulations. To achieve this, we first calculated the three-dimensional mean-squared displacement (MSD),

$$\text{MSD} = \frac{1}{N}\sum_{i=1}^{N}\langle(\vec{r}_i(t) - \vec{r}_i(0))^2\rangle \quad (8)$$

where $\vec{r}_i(t)$ and $\vec{r}_i(0)$ are the position of atom *i* at time *t* and time *0*, respectively. The MSD was calculated for all molecules making up the system and for each of the ions separately to give MSD. From these values, the diffusion coefficients for each ion can be determined:

$$D = \sum_{t\to\infty} \frac{1}{6t} MSD \tag{9}$$

Provided the limiting diffusive behavior of the ions is observed by the end of the simulations. The NVT-MD simulations for 5 ns at 300 K for three systems. To obtain an indication of the statistical accuracy of the MSD, we repeated these simulations three times for each sample. The initial velocities of atoms in each run were randomly distributed. For thermal transport properties, non-equilibrium molecular dynamics (NEMD) simulations were performed to calculate the thermal conductivity of ionic thermoelectric materials, which has been widely employed to study the thermal conductivity of amorphous organic materials. The heat source and heat sink were set as 320 K and 280 K, respectively, using Langevin thermostats, as shown in Supplementary Fig. 21a. A layer of atoms at each end of the simulation cell was fixed in space to prevent the heat flux across the periodic boundaries. Besides, the translational drift of the sample is blocked by the fixed atoms, which helps to extract the temperature profile. The system runs in the NVE ensemble for 1.5 ns to calculate thermal conductivity. A typical temperature profile at the steady-state is shown in Supplementary Fig. 21b. The heat flux (*J*) is calculated by averaging the energy input and output rates from the heat source and sink per unit across the sectional area in Supplementary Fig. 21c. The thermal conductivity (κ) of the system was calculated[8],

$$\kappa = -J/(A \cdot \nabla T) \tag{10}$$

where ∇T is the temperature gradient across the sample. The temperature gradient was obtained by linear fitting to the local temperature, excluding the temperature jumps at the two ends. The

thermal conductivity is averaged for six independent simulations with different initial velocities. The error bars are the standard derivation of these data.

Calculation of the entropy of cation and anion in i-TE systems

The entropy is predicted by the MD simulations according to

$$S = \frac{K_B}{2} lndet(\frac{K_B T e^2}{\hbar^2} \boldsymbol{M}\delta + \boldsymbol{1}) \tag{11}$$

where K_B refers to the Boltzmann constant, $\hbar$ is the reduced Planck constant, e is the Euler's number and T corresponds to the temperature. ***M*** and ***1*** are the mass matrix and the unity matrix, respectively. δ is the covariance matrix of the coordinate fluctuations, with $\delta_{ij} = \langle (x_i - \langle x_i \rangle)(x_j - \langle x_j \rangle) \rangle$[9,10].

Supplementary references

1. Zhao, D., Fabiano, S., Berggren, M. & Crispin, X. Ionic thermoelectric gating organic transistors. *Nat. Commun.* **8,** 14214 (2017).
2. An, M. et al. Predictions of thermo-mechanical properties of cross-linked polyacrylamide hydrogels using molecular simulations. *Adv. Theory Simul.* **2,** 1800153 (2019).
3. Hanwell, M. D. et al. Avogadro: an advanced semantic chemical editor, visualization, and analysis platform. *J. Cheminformatics* **4,** 17 (2012).
4. Dodda, L. S., de Vaca, I. C., Tirado-Rives, J. & Jorgensen, W. L. LigParGen web server: an automatic OPLS-AA parameter generator for organic ligands. *Nucleic Acids Res.* **45,** W331-W336 (2017).
5. Demir, B., Chan, K. Y. & Searles, D. J. Structural electrolytes based on epoxy resins and ionic liquids: a molecular-level investigation. *Macromolecules* **53,** 7635-7649 (2020).
6. Plimpton, S. Fast parallel algorithms for short-range molecular-dynamics. *J. Comput. Phys.* **117,** 1-19 (1995).
7. Martinez, L., Andrade, R., Birgin, E. G. & Martinez, J. M. PACKMOL: A package for building initial configurations for molecular dynamics simulations. *J. Comput. Chem.* **30,** 2157-2164 (2009).
8. An, M. et al. Mass difference and polarization lead to low thermal conductivity of graphene-like carbon nitride (C3N). *Carbon* **162,** 202-208 (2020).
9. Andricioaei, I. & Karplus, M. On the calculation of entropy from covariance matrices of the atomic fluctuations. *J. Chem. Phys.* **115,** 6289-6292 (2001).
10. Karplus, M. & Joseph, N. K. Method for estimating the configurational entropy of macromolecules. *Macromolecules* **14,** 325-332 (1981).
